# Oxygen supplementation in anesthesia can block FLASH effect and anti-tumor immunity in conventional proton therapy

**DOI:** 10.1038/s43856-023-00411-9

**Published:** 2023-12-15

**Authors:** Lorea Iturri, Annaïg Bertho, Charlotte Lamirault, Elise Brisebard, Marjorie Juchaux, Cristèle Gilbert, Julie Espenon, Catherine Sébrié, Laurène Jourdain, Ludovic de Marzi, Frédéric Pouzoulet, Jane Muret, Pierre Verrelle, Yolanda Prezado

**Affiliations:** 1 Institut Curie, Université PSL, CNRS UMR3347, Inserm U1021, Signalisation Radiobiologie et Cancer, Orsay, France; 2 Université Paris-Saclay, CNRS UMR3347, Inserm U1021, Signalisation Radiobiologie et Cancer, Orsay, France; 3 Translational Research Department, Institut Curie, Experimental Radiotherapy Platform, Université Paris Saclay, Orsay, France; 4 INRAE Oniris, UMR703 PAnTher − APEX, Nantes, France; 5 Service Hospitalier Frederic Joliot, CEA, CNRS, Inserm, BIOMAPS Universite Paris-Saclay, Orsay, France; 6 Institut Curie, Université PSL, Université Paris-Saclay, Inserm U1288, Laboratoire de Recherche Translationnelle en Oncologie (LITO), Orsay, France; 7 Institut Curie, Radiation Oncology Department, Campus universitaire, Orsay, France; 8 Institut Curie, Université PSL, Department of Anesthesia and Intensive Care, Paris, France; 9 Institut Curie, Université Paris-Saclay, Inserm U1196, CNRS UMR9187, Chimie et Modélisation pour la Biologie du Cancer (CMBC), Orsay, France

**Keywords:** Paediatric cancer, Paediatric research, CNS cancer, Radiotherapy, Tumour immunology

## Abstract

**Background:**

Radiation-induced neurocognitive dysfunction is a major adverse effect of brain radiation therapy and has specific relevance in pediatric oncology, where serious cognitive deficits have been reported in survivors of pediatric brain tumors. Moreover, many pediatric patients receive proton therapy under general anesthesia or sedation to guarantee precise ballistics with a high oxygen content for safety. The present study addresses the relevant question of the potential effect of supplemental oxygen administered during anesthesia on normal tissue toxicity and investigates the anti-tumor immune response generated following conventional and FLASH proton therapy.

**Methods:**

Rats (Fischer 344) were cranially irradiated with a single high dose of proton therapy (15 Gy or 25 Gy) using FLASH dose rate proton irradiation (257 ± 2 Gy/s) or conventional dose rate proton irradiation (4 ± 0.02 Gy/s), and the toxicities in the normal tissue were examined by histological, cytometric and behavioral analysis. Glioblastoma-bearing rats were irradiated in the same manner and tumor-infiltrating leukocytes were quantified by flow cytometry.

**Results:**

Our findings indicate that supplemental oxygen has an adverse impact on both functional and anatomical evaluations of normal brain following conventional and FLASH proton therapy. In addition, oxygen supplementation in anesthesia is particularly detrimental for anti-tumor immune response by preventing a strong immune cell infiltration into tumoral tissues following conventional proton therapy.

**Conclusions:**

These results demonstrate the need to further optimize anesthesia protocols used in radiotherapy with the goal of preserving normal tissues and achieving tumor control, specifically in combination with immunotherapy agents.

## Plain Language Summary

Proton therapy is a type of precise radiotherapy that can have reduced side effects. Children undergoing proton therapy are often given a general anesthetic, supplemented with high oxygen levels as a measure of safety. However, the consequences of modifying the oxygen concentration in the treatment have not been studied. In this study, we evaluated the consequences of adding oxygen in the anesthesia in a model of brain tumor after conventional proton therapy and a new radiotherapy technique, FLASH proton therapy. We observed that oxygen supplementation can cause more brain damage in FLASH proton therapy and block anti-tumor immune cell infiltration into the tumor in conventional proton therapy. Overall, this study should be taken into consideration when designing new protocols of radiotherapy, specifically those including FLASH proton therapy and combinations with immune-targeted treatments.

## Abstract (web summary)

Iturri, Bertho et al. analyze the effects of oxygen administration during anesthesia in conventional and FLASH proton therapy in a rat model. They demonstrate the detrimental effect of varying oxygen supply using histologic, cytometric and behavioral analysis, and highlighting the urgent need to optimize anesthesia protocols in pediatric oncology.

## Introduction

Radiation-induced side effects, including anatomical and functional deficits, are often observed following fractionated partial or whole-brain irradiation. Follow-up magnetic resonance imaging (MRI) of brain irradiations typically shows vascular and white matter alteration. Focal radiation necrosis is also frequently reported. The development of radiation-induced brain damage can also impair cognitive function, which can occur without apparent anatomical abnormalities^1^. The incidence of radiation-induced neurocognitive dysfunction is likely underestimated due to the low frequency of formal neurocognitive evaluations and the difficulty distinguishing between radiation-induced and tumor-induced side effects. The mechanisms underlying the pathogenesis of radiation-induced memory and attention changes remain poorly understood but are associated with impaired neurogenesis, white matter injury due to oligodendrocyte impairment, vascular lesions, and activation of astrocytes and microglia^2^.

Medulloblastomas, ependymomas, germ cell tumors, low-grade gliomas, and craniopharyngiomas are common pediatric and adult brain tumors that require the application of radiation to large brain volumes for disease control and increased long-term survival^3^. However, consequential cognitive deficits, including deficits in overall cognitive abilities, academic functioning, and specific cognitive skills have been reported in pediatric brain tumor survivors^4^. Consequently, minimizing the risk of late treatment-related adverse effects is necessary.

Almost all children require general anesthesia or sedation to achieve perfectly reproducible patient positioning during radiotherapy or proton therapy^5^. Additionally, general anesthesia is also used in intraoperative electron radiation therapy^6^, but there are currently no published guidelines for the use of iterative anesthesia for radiation therapy (RT). Numerous protocols have been published to date that use a range of airway management procedures^7,8^. Induction of anesthesia can be performed using an open circuit of pure oxygen and hypnotic gas (sevoflurane) administered via a face mask or with intravenous hypnotic agent (propofol) and oxygen delivery via nasal cannula or a face mask (at an oxygen flow rate of 2 L/min)^5,9,10^. Anesthesia can be maintained using intravenous propofol and oxygen via nasal cannula or sevoflurane and oxygen, with more than 50% of the inspired fraction of oxygen administered via a laryngeal or face mask. In all cases, the amount of administered oxygen (O_2_) is non-negligible. However, despite being crucial for pediatric oncology, the potential impact of oxygen administered during anesthesia on the responses of healthy and tumor tissues to radiation has yet to be elucidated.

Proton beam radiation therapy (PT) is one of the most advanced techniques for treating pediatric brain tumors^11^. Many pediatric brain tumors are treated with conventional proton therapy (CPT). However, cranial CPT is associated with adverse neurocognitive outcomes, particularly in younger patients^12^, and its incidence may be underestimated^13^. Although some brain regions are already considered organs at neurocognitive risk during RT, the same regions are reported to be the predominant sites of tumor relapse following RT, particularly the periventricular subependymal area or subventricular zone (SVZ, which contains neurogenesis niches)^14^. Improvements to the therapeutic index of proton therapy are required as consequential cognitive deficits have been reported in pediatric brain tumor survivors. Further, more efficacious, and less damaging therapeutic options are required to minimize the risk of late treatment-related adverse effects while maintaining or increasing tumor control.

Recent developments in radiation therapy modalities have been based on modulation of the physical parameters of irradiation. These innovative RT modalities represent promising therapeutic options for pediatric and radiation-resistant tumors by considerably reducing toxicity in healthy tissues. FLASH radiotherapy (FLASH-RT) is a novel form of RT that involves delivery of radiation at ultra-high dose rates (UHDR, > 40 Gy/s), much higher than currently used in routine clinical practice. Increasing evidence from preclinical studies conducted in the last decade has indicated that UHDR reduce radiation-induced toxicity in the healthy tissues of various organs^15–22^. This net reduction in radiation-induced tissue injury in the context of UHDR has been termed the FLASH effect. FLASH-RT, delivered in fractions or as a single dose, has been reported to lead to equivalent tumor control to conventional RT in glioma-bearing mice^22^, with no cognitive deficits observed after electron FLASH-RT in contrast to conventional irradiation. To date, no extensive evaluation has been performed using FLASH proton therapy (pFLASH).

However, negative results have also been reported from preclinical trials of FLASH-RT^23^, indicating that parameters other than UHDR may contribute to the FLASH effect. These may include beam structure (i.e., pulse width or repetition rate), radiation dose, irradiated volume, and oxygen concentration in radiation-exposed healthy tissue, as the radiobiological mechanisms underlying the FLASH effect remain unknown. One of the hypotheses is that UDHR delivery causes localized oxygen depletion that spares healthy tissues. The critical role of oxygen in sparing normal tissue from the effects of radiation has been reported in cell culture, and mouse models using electron beams^17,24,25^. The initial hypothesis involved the transient formation of hypoxia environments due to oxygen depletion following radiation with UHDR, but it has recently been challenged^26–28^. The role of oxygen concentration in tissues to the FLASH effect remains unclear and warrants further investigation. Accordingly, the aims of the present study were threefold: first, to assess the effect of oxygen administration during anesthesia on brain tolerance after proton therapy; secondly, to determine the potential reduction in neurotoxicity conferred by pFLASH with respect to oxygen concentration; and finally, to evaluate the influence of oxygen in anesthesia on immune cell infiltration into brain tumors. During the course of this study, normal tissue toxicity was evaluated using the therapeutic dose of the glioblastoma model (25 Gy), where pFLASH has already been shown to have a neurocognitive protective effect^29^. Additionally, we decided to further investigate the potential FLASH effect using a lower dose similar to previous electron FLASH studies (15 Gy)^15,17^. To the best of our knowledge, this is the first study of this type to modulate the oxygen concentration in gas anesthesia during both conventional proton therapy (CPT) and pFLASH irradiations. The results presented here show that oxygen supplementation during anesthesia can have deleterious effects on the normal brain following both conventional and FLASH proton therapy as seen by functional and anatomopathological evaluations. In addition, the results evidence that oxygen supplementation is particularly detrimental to antitumor immune responses in conventional proton therapy as it can prevent lymphocyte infiltration into the tumor. These results demonstrate the need to optimize further anesthesia protocols used in radiotherapy that aim to preserve normal tissues and achieve a better tumor control, specifically when combination with immunotherapy agents are planned.

## Methods

All animal experiments were conducted following our institution’s animal welfare and ethical guidelines and were approved by the Ministry of Research (permits no. 2021033117587802 and 2022040609163783). Animals were housed in the Institut Curie animal facility accredited by the French Ministry of Agriculture for performing experiments on rodents. Cages were enriched with cardboard tunnels.

### Irradiation setup and conditions

Irradiation was performed using the “universal” nozzle-equipped gantry at the Orsay proton therapy center (ICPO) using the same setup and dosimetry described in our previous work^29^. Proton irradiation of rats was performed at the plateau region of the Bragg peak curve using standard (4 ± 0.02 Gy/s) and FLASH (257 ± 2 Gy/s) mean dose rates.

Young adult (7-week-old) male Fischer 344 rats (F344, Janvier Labs) received unilateral brain irradiation. Both naive and glioma (RG2)-bearing rats were included in the present study. An in-house 3D-printed rat immobilizer was used^30,31^.

Two types of anesthesia were used in the present study. Half of the rats received isoflurane at a concentration of 2.5% in 21% oxygen (medical air, no oxygen added), hereafter referred to as the “without O_2_”  or “no O_2_” group. The remaining rats were anesthetized with isoflurane delivered in a mixture of medical air and oxygen, with a total oxygen concentration of 70%, hereafter referred to as the “with O_2_” or “O_2_” group.

To assess the long-term effects of proton therapy, 36 naive rats were irradiated with either conventional proton therapy (CPT; *n* = 18) or proton FLASH radiotherapy (pFLASH; *n* = 18). The administered dose was either 25 Gy (*n* = 12 rats “with O_2_”, *n* = 12 rats “without O_2_”, half of them receiving CPT, the others pFLASH), the therapeutic dose for the RG2 glioma model^32^, or 15 Gy (n = 6 rats pFLASH “without O_2_” and *n* = 6 rats CPT “without O_2_”) to assess for potential differences in normal tissue sparing at lower doses. A total of 12 rats were used as non-irradiated controls, half as controls for the 25 Gy series, and the other half as controls for the 15 Gy series. Supplementary Table 1 shows the group distribution.

A total of 35 RG2 tumor-bearing rats were irradiated to investigate radiation-induced immune cell infiltration into tumor tissues and the systemic effects of proton therapy. Rats were divided into five groups and received: CPT with O_2_ (*n* = 5), CPT without O_2_ (*n* = 5), pFLASH with O_2_ (*n* = 7), pFLASH without O_2_ (*n* = 7), and a non-irradiated control group (*n* = 8). Each group was clearly identified on the cage at the time of irradiation. See Supplementary Table 1.

Before experiments, film dosimetry was performed to verify the irradiation conditions. Radiochromic films were also placed on the overlying skin to ensure irradiation quality.

### Tumor inoculation and verification of tumor growth

The RG2 [D74] (ATCC® CRL-2433™) glioma cell line transfected with the luciferase gene and GFP gene was used in the present study (RG2-GFP-luc). A total of 50,000 RG2-GFP-luc cells were suspended in 5 µL DMEM and then intracranially injected into 7-week-old male wild-type rats (strain F344, Janvier Labs) using a Hamilton syringe through a burr hole in the right caudate nucleus (from the bregma: AP: -1; ML: 4 and at a depth of -5.5 mm from the skull).

Tumor growth was confirmed by magnetic resonance imaging (MRI), bioluminescence imaging (BLI), or both methods before irradiation. Bioluminescence imaging (BLI) was done using an IVIS spectrum (PerkinElmer, Houten, the Netherlands) to confirm the presence of a tumor before irradiation. D-luciferin, at a concentration of 150 mg/kg, was injected intraperitoneally and bioluminescence was measured by the IVIS spectrum 25 min later (peak of bioluminescence). The tumor presence was confirmed when the bioluminescence signal overcame the background signal. Rats with significant BLI signal on the day of the irradiation were included in the study and randomized into experimental groups based on the intensity of the signal.

### Animals’ follow up

Naive rats were observed for six months. Behavioral tests were performed one-, three-, and six-months post-irradiation (mpi). Tumor-bearing animals were irradiated 14 days after tumor cell inoculation and sacrificed eight days after irradiation for flow cytometry analysis. Non-irradiated animals were sacrificed 14–16 days after inoculation due to tumor growth. The clinical status of the animals was monitored five times per week, including body weight.

### Magnetic resonance imaging (MRI)

Three or six months after irradiation, naive rats underwent MRI to assess brain injury. MRI images were acquired using a 7-Tesla preclinical magnet with a 35-mm-diameter “bird-cage” antenna (Bruker Advance Horizontal 7-T Bruker, Inc., Billerica, MA). Gadolinium (Gd-DOTA) was administered as a contrast agent at 100 μmol/kg (Guerbet SA, Villepinte, France), via a catheter in the tail vein. Two sequences were acquired: morphological T2-weighted and T1-weighted TurboRare. T1 sequences were acquired before and after the Gd-DOTA injection.

### Oxymetric test

A MouseMonitor (INDUS Instruments) was used to verify the levels of oxygen in the blood during the anesthesia conditions. The animals were anesthesized with isoflurane at a concentration of 2.5% in either only medical air (21% O_2_), a mixture of 50% medical air and 50% oxygen (70% O_2_) or only oxygen (100% O_2_) and positioned on the heating pad of the device. The electrode cream (supplied with the device) was applied to the four legs and taped to the sensors with adhesive tape. The oximetry sensor was placed in the inguinal region

### Assessment of FLASH effect in behavioral tests

Naive rats were housed in groups of two animals per cage in a temperature- and humidity-controlled colony room and maintained on a 12:12 h light/dark cycle with ad libitum access to water and food. The same researcher performed all behavioral tests at approximately the same time each day for each animal to avoid sleep cycle disruption. The open field test (OF) was adopted as a basal assessment to measure locomotor, exploratory activity, and general anxiety. Memory capacity was assessed using the object recognition task (ORT).

In the OF tests, each rat was placed in an open arena (1 m × 1 m) and allowed to explore the environment for 5 min. The total distance travelled, the time spent rearing, and the time spent in the center were recorded. Anxiety was considered inversely correlated with the time the rat spent in the center of the arena. Memory capacity was assessed using the ORT, which evaluates the ability to recognize a novel object in a known environment. To reduce the anxiety related to the exposure of the open field and thus to habituate the animals to this environment, following the OF session of 5 min, animals are placed in the arena for 2 additional sessions of 3 min with an interval of 3 h between each trial. The following day, each rat was allowed to familiarize itself with two identical objects in the OF arena for 5 min. Three hours later, the rat was placed in the OF arena for 5 min with one novel object and the same familiar object. The time spent exploring each object and the total distance travelled were measured and used to calculate the discrimination ratio using the following formula:

$$\frac{{{\mbox{Time}}}\,{{\mbox{spent}}}\,{{\mbox{exploring}}}\,{{\mbox{the}}}\,{{\mbox{novel}}}\,{{\mbox{object}}}\,-\,{{\mbox{Time}}}\,{{\mbox{spent}}}\,{{\mbox{exploring}}}\,{{\mbox{the}}}\,{{\mbox{familiar}}}\,{{\mbox{object}}}}{{{\mbox{Time}}}\,{{\mbox{spent}}}\,{{\mbox{exploring}}}\,{{\mbox{both}}}\,{{\mbox{objects}}}\,}$$

These two tasks were repeated three times (1, 3, and 6 months after irradiation). To avoid learning biases with the repetition of tests, objects presented were always very different from one test to another in term of shape, color, size, and materials.

### Assessment of radiodermatitis in naive rats

The impact of different irradiation modes on the skin was evaluated in naive rats. Skin reactions were scored every two to three days using the following arbitrary scoring system, used in previous studies^33,34^: 0, no changes; 1, dull, faint erythema with epilation; 2, bright erythema with dry desquamation; 2.5, patchy moist desquamation with moderate erythema; 3, confluent moist desquamation with pitting erythema; 4, spontaneous bleeding; 4.5, ulceration; and 5, necrosis. For examples of the grading used in this study see Supplementary Fig. 1.

### Histopathology

At the end of the study period (6 months after irradiation), animals were humanely euthanized by CO_2_ asphyxia. Two animals from the CPT with O_2_ group that received 25 Gy were excluded from the histopathological analysis as one had to be sacrificed due to eye enucleation, and the other died suddenly for no apparent reason.

For histopathological analyses, brains were removed and incubated in Zinc Formalin Fixative following rat necropsy, followed by incubation with 70% ethanol. Brains were then cut into six coronal sections using a method adapted from the Society of Toxicologic Pathology’s guidelines^35^ and embedded in paraffin. 4 µm-thick serial sections were used for hematoxylin and eosin (HE) staining for histopathology evaluations and immunohistochemistry (IHC) to assess microglia (Iba-1, 1:4000, 013–27691, Wako Chemicals, RRID: AB_2934095) and astrocytes (GFAP, 1:2500, Z0334, Dako, RRID: AB_10013382). All histopathological assessments were performed blinded by a board-certified veterinary pathologist.

### Immunochemistry processing

For immunohistochemistry, the tissue sections were deparaffinized and rehydrated prior to antigen retrieval (AR) by heating for 40 min at 98 °C in pH 6 citrate buffer (ZUC028, Zytomed) in a bain-marie. Slides were allowed to cool at room temperature in the AR solution for 20 min and then rinsed. Endogenous peroxidases were blocked by 10 min incubation with 3% hydrogen peroxide at room temperature before washing. For Iba-1 IHC, the slides were then incubated with the Blocking Solution (110, Diagomics) for 30 min at room temperature, followed by incubation for 1 h at 37 °C with the primary antibody diluted in the Blocking Solution. The slides were rinsed and incubated with a Biotinylated Goat Anti-Rabbit IgG antibody (E434, Dako) at a 1:300 dilution for 30 min at room temperature. After washing, slides were incubated with Streptavidin/HRP (P0397, Dako) for 30 min at room temperature diluted at 1:300 dilution. The slides were rinsed prior to incubation with DAB (750118, Invitrogen) until desired staining was obtained. Finally, the slides were washed, counterstained, dehydrated and mounted. For GFAP IHC, no blocking was performed prior to the primary antibody incubation. The primary antibody was incubated for 30 min at room temperature diluted in a solution of 5% normal goat serum in TBS Tween 20. The slides were rinsed and then incubated with a labelled polymer HRP anti-rabbit antibody (Dako EnVision TM + system-HRP (DAB) kit, K4010, Dako; ready to use) for 30 min at room temperature. The slides were rinsed prior to incubation with DAB (750118, Invitrogen) until desired staining was obtained. Finally, slides were washed, counterstained, dehydrated and mounted

### Analysis of peripheral and tumor and brain immune cell populations by flow cytometry

Blood was collected from tumor-bearing rats receiving 25 Gy in tubes with ethylene diamine tetra-acetic acid (EDTA) (OZYME), 24 h and 7 days post-irradiation. Red blood cells were lysed using a Red Blood Cell Lysis Solution (Miltenyi Biotec).

At 8 days post-irradiation, tumors and the contralateral hemisphere of the rat brain were dissected and incubated in digestion solution containing Dulbecco’s phosphate-buffered saline (DPBS), 1 mg/mL collagenase D (Roche), 0.1 mg/mL DNase I (Sigma), and 3% fetal calf serum (FCS). Tissues were then mechanically disrupted with a syringe piston into a 100 μm strain to obtain single cell suspensions in DPBS with 0.5% bovine serum albumin (BSA) and 2 mM EDTA (FACS buffer). Cells were mixed with 30% isotonic Percoll Solution, centrifuged, and blocked with anti-CD32 (FcγRII) blocking agent.

Cells were incubated in a viability stain at a 1:1000 dilution (FVS780, BD Biosciences, RRID: AB_2869673) and immunolabeled in buffer containing PBS and 3% of fetal bovine serum in the case of blood samples, and FACS buffer in the case of tissue samples (Supplementary Table 2). Counting beads were added to each sample before flow cytometry (CountBright™ Plus Absolute Counting Beads, Thermo Fisher). Cell profiles were analyzed using a flow cytometer (Fortessa LSR, BD Bioscience) and FlowJo™ v10.6 Software (BD Life Sciences). Gating strategies are provided in the supplemental materials (Supplementary Figs. 2–4).

### Analysis of systemic cytokines

After blood collection in a heparin tube, the whole blood was centrifugated at 4 °C at 1500 *g* for 15 min, and plasma was separated. Cytokines were measured using chemiluminescence-based V-plex Proinflammatory Panel 2 rat Kit (MSD, K1559D, RRID: AB_2916285).

### Statistical analysis and reproducibility

Radiation dermatitis scoring was analyzed using Two-way ANOVA. Data for peripheral immune cells were compared using the Brown–Forsythe test of variance (One-way ANOVA) with multiple comparisons performed by unpaired t with Welch’s correction. Data regarding intratumoral immune cell infiltration and circulating cytokine levels were compared using one-way ANOVA or two-way ANOVA with multiple comparisons using uncorrected Fisher’s LSD test. Statistical analyses were performed using GraphPad Prism 9 software (GraphPad Software, CA, United States).

Behavioral tests were analyzed using Student’s t-test in JASP software with a threshold of 0.05. Bayesian independent sample t-tests were further conducted to characterize the findings for comparisons with a non-significant result. The sample size was determined using G*power software. The behavior studies presented in this article have not been replicated due to the limited access to the proton beam line for research. Concerning the studies on tumor-bearing rats, they were carried out in 3 independent experiments.

### Reporting summary

Further information on research design is available in the Nature Portfolio Reporting Summary linked to this article.

## Results

### Influence of oxygen content on skin toxicity

**Fig. 1:**
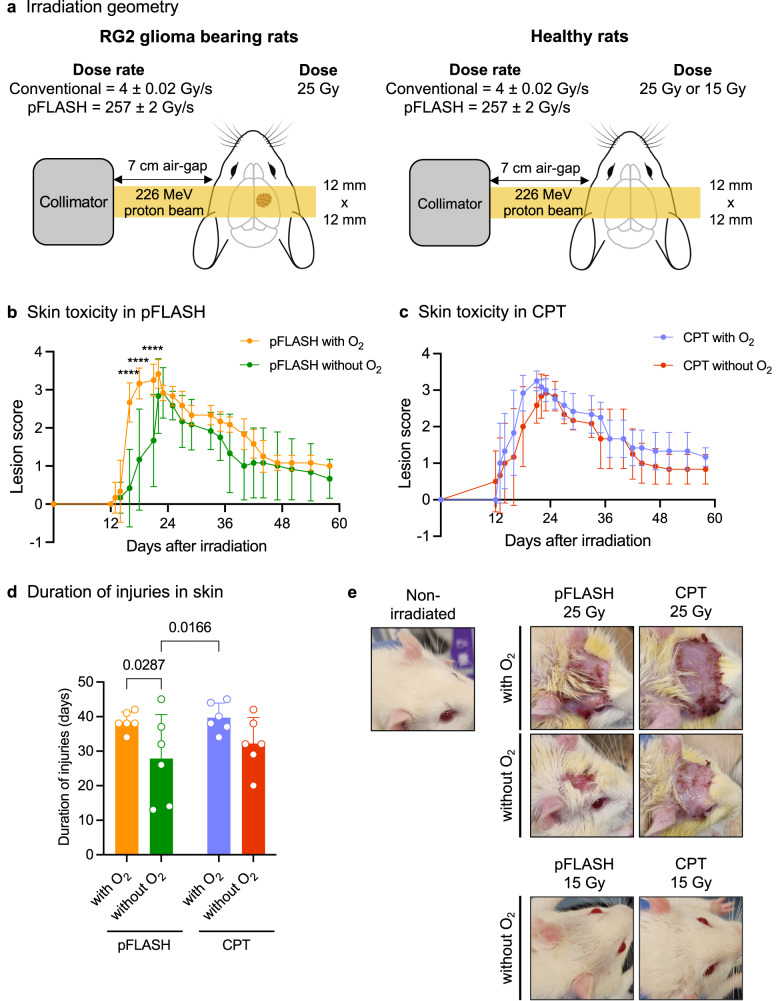
Impact of oxygen content on the development of radiation dermatitis after pFLASH and CPT delivering 25 Gy. **a** Irradiation geometry. All groups received unilateral transmission irradiations using the plateau area of a mono energy 226 MeV proton beam. The original scanned beam was modified into a 12 × 12 mm² collimated scanned beam at the irradiation point using a brass collimator (and 7 cm airgap), with a flatness of §5% at maximum dose level. In all cases, the dose prescription was 25 Gy at 1 cm depth in the brain or in the tumor. **b** Average lesion score after pFLASH irradiation as a function of oxygen level (*n* = 6 in each group). **c** Average lesion score after CPT, as function of oxygen level (*n* = 6 in each group). **d** Duration of injuries among the irradiated groups (*n* = 6 in each group). **e** Representative images of radiation dermatitis among the groups. Images in the groups receiving 25 Gy (*n* = 6 in each group) at 22 days post-irradiation (maximal severity), correspond to the mean grade observed on this day (grade 3 for pFLASH with O_2_, grade 2.5 for pFLASH without O_2_, grade 3 for CPT with O_2_, grade 3 for CPT without O_2_). The rats receiving 15 Gy in CPT or pFLASH did not develop radiation-induced injuries. The data are presented as the mean ± standard deviation (SD).

In this study, rats were irradiated with a 226 MeV proton beam cranially (Fig. 1a) under anesthesia. The anesthesia gas was administered either mixed with medical air (experimental groups “without O_2_” or “with O_2_”, which changed the oxygen saturation in the blood during the irradiation time (Supplementary Fig. 5). With regard to healthy rat analysis, all irradiated animals that received 25 Gy developed radiation dermatitis starting 12 to 22 days after irradiation. Globally, no significant differences in average lesion scores were observed between groups (Fig. 1b, detailed statistics available Supplementary Table 3, Supplementary Fig. 6, and Supplementary Table 4). It is noted that despite the oxygen levels in anesthesia did not statistically influence the development of radiation dermatitis in the pFLASH group (overall *p*-value = 0.0569), a significant difference in severity was observed at 16 days post-irradiation (dpi; *p*-value < 0.0001), 18 dpi (*p*-value < 0.0001) and 21 dpi (*p*-value = 0.0002). Therefore, this difference was specific to the time of the highest toxicity grade.

The duration of radiodermatitis was significantly shorter in the pFLASH without O_2_ group compared to the pFLASH and CPT with O_2_ groups (Fig. 1c, *p*-value = 0.0287), indicating tissue oxygen content following pFLASH irradiation affected the duration of radiodermatitis. In contrast, oxygen concentration was not significantly associated with the duration of radiation dermatitis in the CPT group (p-value = 0.2422). Representative images of radiodermatitis lesions are presented in Fig. 1d. See Supplementary Fig. 1 for illustrated scoring.

None of the animals that received 15 Gy developed skin injury regardless of the irradiation mode and oxygen concentration (Fig. 1d, right panel).

### Influence of oxygen content on brain toxicity

MRI images were acquired six months post-irradiation (mpi) after irradiation among the 25 Gy groups, with Gadolinium injections as a contrast agent to observed blood-brain barrier (BBB) lesions on T1-weighted images. MRI images revealed the presence of lesions in rats in the pFLASH with O_2_ group (Supplementary Fig. 7). Three out of six animals had extensive lesions compatible with radiation necrosis in the fimbria and fornix of the hippocampus and BBB breakdown in the same region, while no major injuries were observed in the rest of the groups. These MRI findings indicate greater brain injury in the pFLASH with O_2_ group. This was not observed in the groups treated with 15 Gy (Supplementary Fig. 8). Anatomopathological analyses, including histology analysis and immunohistochemistry, revealed no signs of brain injury in rats that received 15 Gy (Supplementary Fig. 9). Thus, no difference in brain toxicity was revealed between pFLASH and CPT, with no effect of the oxygen supplementation observed at this irradiation dose. Of the 22 animals that received 25 Gy using either irradiation mode, 19 had microscopic injuries on anatomopathological analysis (Supplementary Fig. 10). In all groups, the nature and localization of the lesions were identical regardless of the irradiation mode and oxygen concentration administered during anesthesia.

As the microscopic lesions were similar between irradiation modes, a scoring method was developed to underline the varying intensity of the observed lesions. The microscopic lesion scores for each group are presented in Table 1. The most severe lesions were observed in the pFLASH with O_2_ and CPT without O_2_ groups, with, respectively, 5 out of 6 animals presenting radiation necrosis on the hippocampus. pFLASH without O_2_ was the group presenting the least neurotoxicity, with 2 animals presenting no histologic changes and only 3 animals presenting hippocampal radiation necrosis.

**Table 1:** Histopathological scoring of the irradiation-induced lesions observed in the 25 Gy series.

Groups	Number of animals	Score		
		0	1	2
Control	6	6	0	0
pFLASH with O_2_	6	0	1	5
pFLASH without O_2_	6	2	1	3
CPT with O_2_	4	0	2	2
CPT without O_2_	6	1	0	5

Score 0: no microscopic lesion, score 1: early and limited lesions (sign of degeneration observed in the optic tract and histopathologic changes in the choroid plexuses), score 2: lesion of radiation necrosis (mainly, necrosis centered on the fornix system of the hippocampus).

Oxygen supplementation during anesthesia was associated with higher toxicity in both irradiation modes. Brain toxicity was exacerbated by oxygen supplementation in CPT and pFLASH groups, where all animals presented severe microscopic lesions. In contrast, two animals of the pFLASH without O_2_ group and one animal of the CPT without O_2_ group had no microscopic sign of brain injury. This deleterious effect of oxygen seemed more relevant for pFLASH than CPT. Interestingly, increase of microglial marker Iba-1 and astroglial marker GFAP were only associated to necrotic lesions (Supplementary Fig. 10) and no differences were observed in the hippocampus or its subregions such as the subventricular zone, Cornu Ammonis 3 or dentate gyrus (DG) (data not shown).

**Fig. 2:**
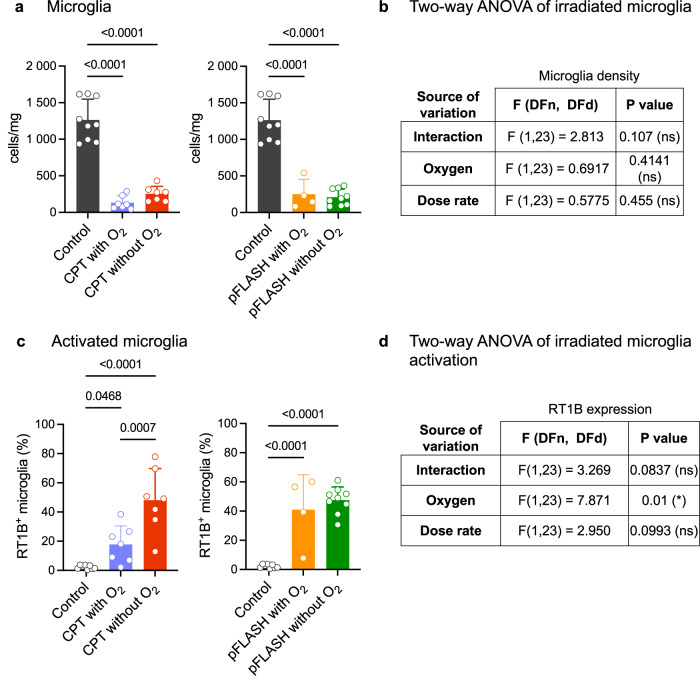
Phenotype of brain microglia 8 days after the irradiations by flow cytometry. **a** Density of microglia in the brain parenchyma compared in the left panel to conventional proton therapy with oxygen added in the anesthesia (purple, *n* = 7) or no oxygen added (red, *n* = 7), and on the right panel, compared to pFLASH with oxygen added in the anesthesia (orange, *n* = 4) or no oxygen added (green, *n* = 9), and non-irradiated controls (black, *n* = 9). **b** Summary of statistical analysis of irradiated samples by two-way ANOVA regarding microglia density in the brain. **c** Proportion of activated microglia by the expression of RT1B. **d** Summary of statistical analysis of irradiated samples by two-way ANOVA regarding proportion of RT1B expression in microglia. The data are presented as the mean ± standard deviation (SD).

The analysis of microglia abundance and microglial expression of the activation marker RT1B (antigen-presenting MHC class II molecule) provided insight into the neuroinflammation triggered by the irradiation modes and oxygen levels over a shorter time interval. We previously demonstrated that microglial brain density decreases in a short timepoint after brain irradiation both with CPT and pFLASH and expresses higher levels of RT1B^29^. Figure 2 shows the effect of oxygen supplementation in microglial population by flow cytometry analysis eight days after irradiation in glioma-bearing rats treated with different irradiation modes. The oxygen supplementation administered during anesthesia did not affect total microglia density, which remained affected in all irradiated groups (Fig. 2a, b). However, the microglia of rats following CPT and oxygen supplementation expressed less RT1B than the group with no oxygen supplementation, while oxygen supplementation during anesthesia did not affect microglial activation following pFLASH (Fig. 2c). The statistical analysis by two-way ANOVA of the irradiated samples showed that oxygen supplementation was the parameter that predominantly impacted microglia activation after radiation (Fig. 2d, *p*-value = 0.01).

### Impact of oxygen supplementation during anesthesia on motor, emotional, and cognitive functions of healthy rats after pFLASH and CPT irradiation

No significant difference in motor, emotional, and cognitive functions was observed between the 15 Gy irradiated groups (Supplementary Fig. 11, detailed statistics are available in Supplementary Note 1). In the case of animals irradiated with 25 Gy, even if some significant differences were observed between the groups, globally, all the animals had correct locomotor (Supplementary Fig. 12 and Supplementary Note 2), and exploratory activity (Supplementary Fig. 13 and Supplementary Note 3) during the time of observation, and no clinical signs of motor alteration were observed.

However, a marked effect of oxygen concentration during anesthesia on cognitive function was observed in rats that received 25 Gy using both irradiation modes and between the CPT without O_2_ and pFLASH without O_2_ groups.

**Fig. 3:**
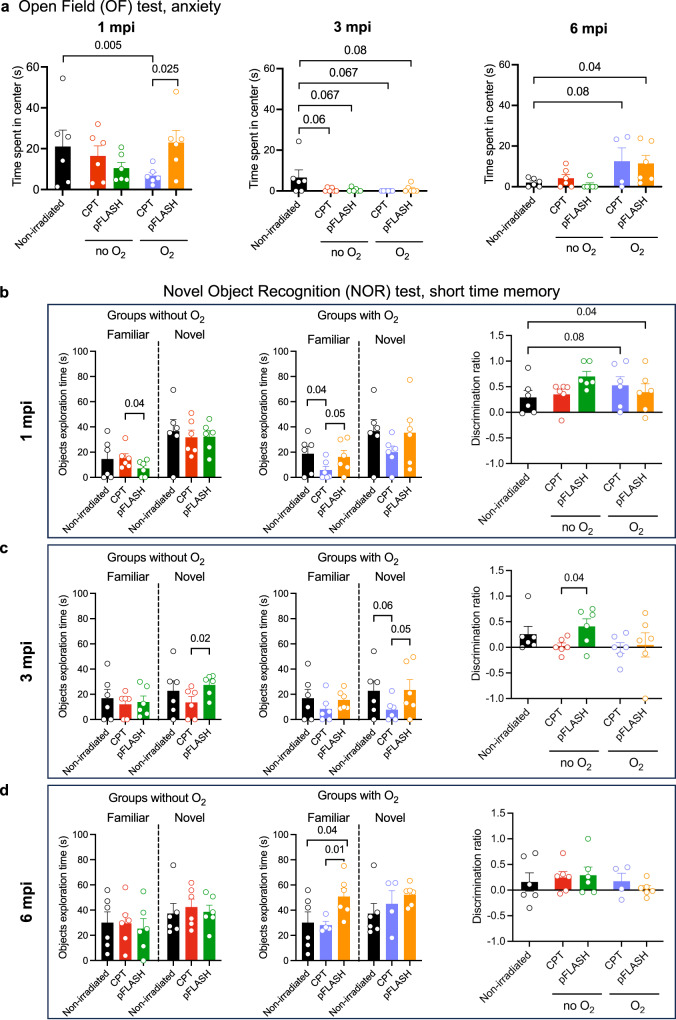
Results of behavioural assays to assess cognitive toxicity. **a** Comparison of anxiety in 5 min open field test and **b** novel object recognition test (ORT) 1 month after treatment, **c** 3 months after treatment, and **d** 6 months after treatment in rats with no irradiation (Controls, black, *n* = 6) and rats receiving 25 Gy in conventional dose rate with oxygen (purple, *n* = 6) or without oxygen (purple, *n* = 6), or in high dose rate with oxygen (orange, *n* = 6) or without oxygen (green, *n* = 6) with or without oxygen supplementation (O_2_) during anesthesia. The data are presented as the mean ± standard error of the mean (SEM). mpi = months post-irradiation.

Anxiety was measured as time spent in the center of the arena (Fig. 3a), since the time spent in the center is inversely correlated to the anxiety state of the animal. Of the animals irradiated with supplemental oxygen during anesthesia, rats in the CPT group had greater anxiety from the first month after irradiation (Control *vs* CPT O_2_: t(10) = −1.75, *p* = 0.055; BF-0 = 2.05: in favor of the alternative hypothesis; and pFLASH O_2_ *vs* CPT O_2_: t(10) = −2.63, *p* = 0.025; BF10 = 2.8: in favor of the alternative hypothesis). In general, all irradiated animals exhibited higher anxiety levels three months after irradiation regardless of the irradiation mode or the supplementation of oxygen during anesthesia (Control *vs* CPT no O_2_: t(10) = −1.63, *p* = 0.067; BF-0 = 1.8: in favor of the alternative hypothesis; Control *vs* pFLASH no O_2_: t(10) = 1.64, *p* = 0.067; BF-0 = 1.8: in favor of the alternative hypothesis; Control *vs* CPT O_2_: t(5) = 1.8, *p* = 0.067; BF + 0 = 1.9: in favor of the alternative hypothesis; Control *vs* pFLASH O_2_: t(10) = 1.5, *p* = 0.08; BF + 0 = 1.6: in favor of the alternative hypothesis).

On the other hand, short-term memory is measured with the novel object recognition test (NOR, Fig. 3b–d and Table 2). One month after the irradiation, all groups had the correct discrimination ratio (discrimination ratio > 0, Fig. 3b and Table 2), and the greater discrimination ratio observed in the pFLASH without O_2_ (pFLASH no O_2_ *vs* control: t(10) = −2.4, *p* = 0.04; BF10 = 2.2: in favor of the alternative hypothesis; pFLASH no O_2_ *vs* CPT no O2: t(10) = −2.45, *p* = 0.03; BF10 = 2.3: in favor of the alternative hypothesis) was due to the lower familiar object exploration time (CPT no O_2_ *vs* pFLASH no O_2_: t(10) = 1.9, *p* = 0.04; BF + 0 = 2.36: in favor of the alternative hypothesis) and not to a difference in the novel object exploration time. At 3 months post-irradiation, only the rats in the control and the pFLASH without O_2_ had correct discrimination ratio, and the object exploration time data confirmed that CPT with and without O_2_ had a lower novel object exploration time (Fig. 3c, CPT no O_2_ *vs* pFLASH no O_2_: t(10) = −2.4, *p* = 0.02; BF-0 = 4.07: in favor of the alternative hypothesis; CPT O_2_ *vs* control: t(10) = −1.7, *p* = 0.06; BF-0 = 2: in favor of the alternative hypothesis; CPT O_2_ *vs* pFLASH O_2_: t(10) = −1.8, *p* = 0.05; BF-0 = 2.1: in favor of the alternative hypothesis). Finally, at 6 months post-irradiation all groups showed a correct discrimination ratio except the pFLASH with O_2_ and the control groups explained by the greater but similar both familiar and novel object exploration time of the rats in the pFLASH with O_2_ group (Fig. 3d, Table 2).

**Table 2:** One sample T-test and Bayesian sample T-test of CPT and pFLASH, with or without oxygen groups to evaluate discrimination ratio compared to 0.

Novel object recognition (NOR)				
	1 mpi	3 mpi	6 mpi	Recognition memory
Non-irradiated controls	t(5) = 2.1, p = 0.04 (*); B + 0 = 2.63 (*) = superior to 0	t(5) = 1.7, *p* = 0.07 (ns); B + 0 = 1.73 (*) = superior to 0	t(5) = 0.9, *p* = 0.2 (ns); B + 0 = 0.8 = not superior to 0	Altered at 6 mpi
CPT without O_2_	t(5) = 3.4, *p* = 0.01 (**); B + 0 = 7,99 (*) = superior to 0	t(5) = 0.4, *p* = 0.3 (ns); B + 0 = 0.5 (ns) = not superior to 0	t(5) = 2.17, *p* = 0.04 (*); B + 0 = 2.75 (*) = superior to 0	Transient memory impairment at 3 mpi
CPT O_2_	t(5) = 3.1, *p* = 0.01 (**); B + 0 = 6.6 (*) = superior to 0	t(5) = 0.1, *p* = 0.5 (ns); B + 0 = 0.3 (ns) = not superior to 0	t(5) = 1.13, *p* = 0.17 (ns); B + 0 = 1.7 (*) = superior to 0	Transient memory impairment at 3 mpi
pFLASH without O_2_	t(5) = 7.1, *p* < 0.001 (***); B + 0 = 92,4 (*) = superior to 0	t(5) = 2.75, *p* = 0.02 (*); B + 0 = 4.75 (*) = superior to 0	t(5) = 1.83, *p* = 0.06 (ns); B + 0 = 2 (*) = superior to 0	Not altered
pFLASH O_2_	t(5) = 2.4, *p* = 0.03 (*); B + 0 = 3.56 (*) = superior to 0	t(5) = 0.2, *p* = 0.4 (ns); B + 0 = 0.4 (ns) = not superior to 0	t(5) = 0.5, *p* = 0.3 (ns); B + 0 = 0.9 (ns) = not superior to 0	Permanent memory impairment from 3 mpi

Only pFLASH without O_2_ animals had non-altered recognition memory over the months post-irradiation.

Overall, rats that had received CPT had transient amnesic alterations at three months, regardless of the concentration of supplemental oxygen during anesthesia. Rats in the pFLASH with O_2_ group also had permanent mnesic alterations from three months onward, but this was not the case in the pFLASH without O_2_ group, which had no mnesic alterations. Thus, only rats in the pFLASH without O_2_ had a correct and unaltered recognition memory.

Specifically, the analysis over the months gave critical information with regard to long-term memory (Supplementary Tables 5–7). Indeed, the decrease in the distance traveled and the time spent to the rear is a good indicator of memorizing the context over time. Rats in the control and pFLASH without O_2_ groups had a normal diminution of locomotor activity as a function of time. In contrast, memorization of context was altered in rats in the pFLASH with O_2_ group starting from three months after irradiation and at six months after irradiation in rats in the CPT with and without O_2_ groups. Similarly, normal reduction in exploratory activity with time was observed in rats in the controls and pFLASH without O_2_ groups. In contrast, there was no reduction of exploratory activity in the pFLASH and CPT with O_2_ groups at six months post-irradiation and from 3 months after irradiation in rats in the CPT without O_2_ (Supplementary Fig. 13 and Supplementary Table 6). In addition, a normal diminution in the time spent in the center of the area as function of time was observed in all groups except CPT and FLASH with O_2_ groups (Fig. 3a and Supplementary Table 7), indicating altered memorization of context at three and six months after irradiation, respectively.

Importantly, these findings indicate that only the pFLASH without oxygen supplementation in anesthesia condition preserved memorization of the spatial context over a long time period (6 months), suggesting that the protective effect of pFLASH in neurocognition is lost by supplementation of oxygen in the anesthesia gas.

### Tumor-infiltrating immune cells, circulating immune cells, and cytokine levels in glioma-bearing rats

**Fig. 4:**
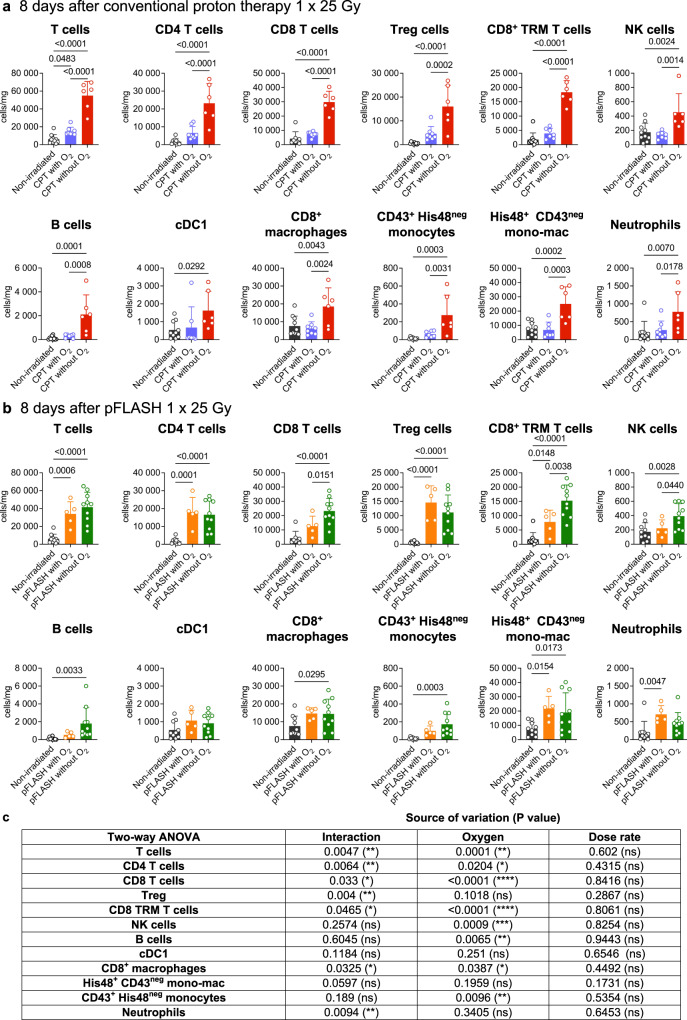
Flow cytometry analysis of immune cells in RG2 glioblastoma. **a** 8 days after conventional proton therapy (CPT), delivering 25 Gy, with (purple, *n* = 7) or without O_2_ aspiration during anesthesia gas (red, *n* = 6), and **b** pFLASH irradiations, delivering 25 Gy, with (orange, *n* = 5) or without O_2_ (green, *n* = 10) aspiration during anesthesia, and non-irradiated control (black, *n* = 10). Quantification of the cell density as recovered cells per milligram of tumor, including all T cells, CD4 T cells, CD8 T cells, regulatory T cells (Tregs), CD8^+^ tissue-resident memory (TRM) T cells, NK cells, B cells, cDC1, CD8^+^ macrophages, CD43^+^ His48^neg^ monocytes, His48^+^ monocytes-macrophages (mono-mac) and neutrophils. **c** Summary of two-way ANOVA statistical analysis of only irradiated tumors. The data are presented as the mean ± standard deviation (SD).

A significant infiltration of immune cells in the tumor microenvironment was observed following CPT irradiation with no oxygen supplementation (Fig. 4a). Greater numbers of T cells and T cell subtypes like CD4 T cells, CD8 T cells, regulatory T cells, CD8^+^ tissue-resident memory (TRM) T cells, as well as NK cells, B cells, CD8^+^ macrophages, neutrophil and monocytic populations, were observed in the CPT group with no supplemental oxygen during anesthesia compared to the non-irradiated controls. However, the rat tumors receiving oxygen supplementation during the anesthesia did not have an increase of any cell type analyzed compared to non-irradiated controls (Fig. 4a). Conventional proton therapy significantly increased tumor-infiltrating lymphocytes (TILs) when no oxygen supplementation was introduced, with respect to oxygen supplementation during anesthesia (p-value of multiple comparisons CPT with O_2_ *vs* CPT without O_2_: T cells *p*-value < 0.001, NK cells *p*-value = 0.0014 and B cells *p*-value = 0.008).

On the other hand, both pFLASH groups had a significant increase in tumor infiltrating leukocytes except for conventional dendritic cells type 1 (cDC1). Overall, oxygen concentration during anesthesia in pFLASH groups had lower effect on the immune cell infiltration into tumor tissues, except for a greater number of infiltrating CD8 T cells in rats that received no supplemental oxygen during anesthesia (*p*-value of multiple comparisons pFLASH with O_2_ *vs* pFLASH without O_2_ *p*-value = 0.0151), CD8^+^ TRM T cells (*p*-value = 0.0038) and NK cells (*p*-value = 0.0440) (Fig. 4b). Further statistical analysis of irradiated samples to decipher the impact of dose rate or oxygen supplementation in tumor immune cell increase showed that the oxygen concentration was the parameter that most significantly influenced tumor immune populations (Fig. 4c). Importantly, all subtypes of T cells analysed were influenced by the interaction between dose rate and oxygen.

The effect of supplemental oxygen during anesthesia in TILs was thus predominantly observed in conventional proton therapy, indicating that inflammatory cell infiltration following CPT is more sensitive to tumor oxygenation than following pFLASH.

The concentration of peripheral blood pro-inflammatory cytokines was monitored in glioma-bearing rats receiving 25 Gy 24 h, and 7 days post-irradiation. No differences in serum levels of IL-6, CXCL1, or TNF-α were detected between groups at 24 h and seven days post-irradiation (Supplementary Fig. 14). However, serum IFN-γ concentration was increased 24 h post irradiation (hpi) in the CPT without O_2_ group (*p*-value = 0.0096) and in the pFLASH with O_2_ group at 24 hpi (*p*-value = 0.0352) that was maintained at 7dpi (*p*-value = 0.0311). Serum IL-10 concentration was transiently increased in all irradiated groups at 24 hpi compared to the control group and maintained in the pFLASH without O_2_ group at seven days post-irradiation.

Oxygen supplementation in anesthesia impacted the concentration of peripheral blood cells specially after pFLASH, compared to the non-irradiated controls and CPT (Supplementary Fig. 15). Oxygen supplementation during pFLASH irradiations decreased the proportion of CD4 T cells at 24 hpi and 7dpi (detailed statistics available in Supplementary Fig. 15h). In the myeloid lineage, pFLASH with O_2_ increased circulating monocyte proportion (both CD43^+^ monocytes at 7 dpi and His48^+^ monocytes at 24 hpi, and neutrophils at 7 dpi), suggesting an increase in the inflammatory response.

## Discussion

The present study was designed to determine the effect of oxygen supplementation during anesthesia and radiation dose rate on normal tissue tolerance and anti-tumor immunity in brain proton therapy. This is a particularly relevant clinical question in pediatric oncology and our study is the first comprehensive preclinical evaluation of the impact of this parameter along with ultra-high dose rate proton therapy in normal and tumoral tissue.

Proton therapy has the advantage of offering better healthy tissue sparing. Therefore, proton therapy has an important place in the therapeutic arsenal of pediatric brain tumours. This study has been carried out at the same proton beam line used to treat pediatric medulloblastoma, requiring whole brain irradiations. The present study compared conventional proton therapy to an innovative radiotherapy technique, pFLASH. FLASH-RT has been reported to reduce the sequelae of brain radiotherapy in young rodents^16,17,29,33,36^, albeit mostly in electron beams. Most children require general anesthesia or sedation to achieve perfect reproducible positioning during radiation therapy. However, there remains a lack of consensus regarding optimum airway management with numerous protocols in current use. The cognitive functions of children undergoing repetitive anesthesia procedures have been extensively evaluated^34^. Nevertheless, to the best of our knowledge, no previous studies have assessed the potential impact of supplemental oxygen during anesthesia neither in conventional radio or proton therapy nor in pFLASH treatments on anatomical and cognitive function and immune response to treatment.

Supplemental oxygen during anesthesia or sedation is essential as small children have an increased oxygen consumption and a reduced functional residual capacity, which leads to a more rapid desaturation than in adults in the case of apnea^37^. In addition, general anesthesia is frequently associated with relative central or obstructive hypoventilation also requiring large amounts of oxygen to maintain normal arterial oxygen saturation levels. Maintaining a high level of oxygen saturation remains the greatest margin of safety in the event of unexpected airway obstruction and sudden desaturation. This gives a margin of time for the practitioner to cover the distance to the child in cases of emergency through the automatic door inside the bunker, which is most often located at a distance from the control station.

In the present study, the animal treated with pFLASH without oxygen supplementation had the lowest degree of brain injury. Despite the reduced side effects observed, brain tissue damage was not negligible following the administration of 25 Gy, the therapeutic dose for gliomas in this animal model^32^. All animals developed radiodermatitis, with 50% of animals developing radionecrosis and microglial activation. Of note, all animals exhibited anxiety, which contrasts other novel techniques such as proton minibeam radiation therapy (pMBRT), where no skin damage, microglial activation, or cognitive or emotional sequelae are observed following administration of the same dose^31,38^. This finding indicates that pMBRT may represent a safer option for neuro-oncology treatments.

On the other hand, the configuration leading to the highest level of brain injury according to anatomopathological evaluations, MRI, and behavioral tests was pFLASH with supplemental oxygen during anesthesia. However, the low number of animals in the CPT with O_2_ group for brain toxicity warrants a cautious interpretation of the results. Thus, caution must be taken when administering ultra-high dose rates in anesthetized patients. Compared to conventional radiotherapy techniques, more severe damage can be induced, even in well-oxygenated normal tissues.

Contrary to electron FLASH beams, where dose escalation studies have been reported^17,18^, systematic dose escalation studies that show the impact of increasing single-dose pFLASH on brain function are lacking. The limited proton beam time access in a clinical facility is a major limitation in these studies. We therefore chose to assess whether the neuroprotective FLASH effect can be triggered at a lower dose. However, 15 Gy did not trigger any evident radiation-induced injuries and proved too low a dose to observe the FLASH effect in the rat animal model. Indeed, the lower dose of 15 Gy did not allow discrimination between groups, as no lesions or side effects were observed in the histopathological and neurotoxicity evaluations.

Overall, pFLASH irradiations resulted in a significant memory sparing compared to conventional proton irradiation, whereas the supplementation of oxygen in anesthesia gas had a detrimental effect on recognition memory after both CPT and pFLASH irradiation, which persisted 6 months post-irradiation in the case of pFLASH. Our observations are consistent with previously published data in electron FLASH, where oxygen supplementation via carbogen breathing suppressed the protective FLASH effect on cognitive function (NOR behavior) 2 months post-irradiation^17^. Previous work demonstrated a four-fold decrease in microglial density following CPT and pFLASH without supplemental oxygen during anesthesia compared to non-irradiated controls^29^. This work showed no difference observed with the use of oxygen supplementation during anesthesia. However, the use of higher concentrations of oxygen during CPT did diminish microglial activation.

Additionally, this study also showed a previously unknown interrelation between oxygen saturation, dose rate, and radiation-induced immune response to the tumor. Generally, higher oxygen content prevented an efficient tumor immune infiltration into the tumor which was specially significant following CPT. However, tumoral infiltration of immune cells following pFLASH appeared to be less affected by supplemental oxygen during anesthesia. TIL infiltration following CPT appeared particularly more sensitive to tumor oxygenation than pFLASH, as no significant lymphocyte increase was observed in CPT without oxygen supplementation while there was no difference in pFLASH groups. This might indicate different inflammatory/immune response pathways involved in CPT and pFLASH, likely related to classical NF-κB and HIF-1 pathways that are linked to oxygen availability^39^.

The detrimental effect of supplemental oxygen during CPT and pFLASH challenges the so-called oxygen depletion hypothesis of FLASH that is currently being discussed in the literature^27^. Although higher partial pressure of oxygen was also detrimental to short-time memory after a preclinical model of electron FLASH^17^, we also observed an increased anxiety in the groups irradiated with O_2_ in the anesthesia gas The significant difference seen in NOR between FLASH groups with or without carbogen breathing in this paper could be explained by many factors, including the content of oxygen in the gas preparation, the energy source or the therapeutic dose delivered in this manuscript. However, we also confirmed the deleterious effect of O_2_ by long-term context memory evaluation as well as skin and brain toxicity. Therefore, we confirmed that the deleterious role of oxygen pressure in the FLASH effect observed in electrons is conserved in protons in a clinically available setting and provided multiple biological observations of this effect. The novelty of this study relies on the analysis in a clinical proton beam setting, a comprehensive evaluation of this effect, and, importantly, the determination of previously unknown correlation between dose rate and tumor immune infiltration blockage dependent on oxygen pressure at the time of irradiation.

One limitation of the present study is that no oxidative parameters were experimentally monitored. Indeed, since the brain is a phospholipid-rich organ, an alternative explanation may be an increased probability in FLASH dose rates of bimolecular recombination of fatty acids that have lost a hydrogen ion from the OH radical (O• carbon centered radical), with an excess of oxygen favoring the formation of ROO• peroxyl radicals instead. Accordingly, lipid peroxidation of phospholipids in the cellular plasma, organelle membrane, or mitochondrial compartment has been shown to alter cell signaling, dysfunction, or death^40,41^. Lipid peroxidation also appears to be involved in brain aging^42,43^. On the other hand, previous studies have reported no lipid peroxidation following FLASH^44^. In this respect, lipid peroxidation reactions that consume oxygen warrant further scrutiny as a possible mechanism underlying the FLASH effect^45^. Finally, the distinct free radical production and recombination after pFLASH or CPT might activate some other pathways related to ROS-mediated immunomodulation^46^ and could explain the differences in tumor immunity. Therefore, future studies focusing on brain proton therapy should investigate these parameters.

Oxygen administration is mandatory to ensure the safe delivery of anesthesia. However, the concentration of oxygen administered is never discussed in anesthetic practice, given the absence of deleterious effects outside of neonates and the overriding safety issue, particularly in this complex setting. A PET scan study following hypoxia radiotracers indicated that the tumor is not more oxygenated when mice breathe 100% oxygen in a subcutaneous tumor model. However, the tumor in this model could have a different oxygen availability than the orthotopic brain tumor used in the present study^47^.

If these results are confirmed in humans by further studies, the benefits and risks of administering high or low oxygen concentrations during anesthesia will need to be discussed. In some procedures, such as laser treatment of ENT (ear, nose, and throat) lesions, it is necessary to ventilate patients with a gas mixture containing less than 30% oxygen due to the risk of fire. The results of the present study may prompt changes to protocols for general anesthesia or sedation in children receiving brain radiotherapy or proton therapy since its findings highlight the detrimental effect of oxygen supplementation during anesthesia on CPT and pFLASH irradiations, particularly following pFLASH, where the most extensive brain injury is observed, and also on the anti-tumor immune response.

## Conclusions

This study presents the first comprehensive preclinical evaluation of anesthesia oxygen impact in proton therapy. The neuroprotective effect of FLASH therapy has been extensively confirmed in proton beams for the first time. However, detrimental effects of oxygen supplementaction in the anesthesia gas were observed both in conventional and FLASH beams, more deleterious in the latter case. Importantly, this study demonstrates that oxygen supplementation in conventional proton therapy hinders tumoral infiltration by immune cells while this parameter is not as influential in proton FLASH radiotherapy, further suggesting that radiation-induced immune regulatory pathways are susceptible to the proton beam dose rate.

These observations should be taken as a word of caution to revise the current anesthesia protocols and may facilitate the development of novel anesthesia protocols to reduce the detrimental effects of radiotherapy while preserving or improving anti-tumor efficacy. The results presented here should also be considered when designing novel radio-immunotherapy combinations involving proton therapy in patients anesthetized during irradiations.

### Supplementary information

Supplementary Information

Description of Additional Supplementary Files

Supplementary Data 1

Reporting Summary

## Data Availability

The raw data of flow cytometry is in the form of separate FCS files that are available from the corresponding author, YP, upon reasonable request. Source data are available as Supplementary Data 1.
