# Supplementary material for: Oxygen supplementation in anesthesia can block FLASH effect and anti-tumor immunity in conventional proton therapy
Source: Commun Med (Lond). 2023 Dec 15;3:183. doi: 10.1038/s43856-023-00411-9 (PMC10724215; doi:10.1038/s43856-023-00411-9)
Supplement: Supplementary file 1 — Supplementary Information [file 43856_2023_411_MOESM1_ESM.pdf]

Supplementary information

Supplementary material and methods

Groups distribution

Rats	Dose	CPT with O_2	pFLASH with O_2	CPT without O_2	pFLASH without O_2	Non-irradiated controls
Naïve	25 Gy	6	6	6	6	6
	15 Gy	N/A	N/A	6	6	6
RG2-bearing	25 Gy	5	5	7	10	8

Supplementary Table 1. Distribution of the experimental groups

Analysis of peripheral, brain and tumor immune cell populations by flow cytometry

Antibody	Fluoro-chrome	Clone	Dilution	Identifier	Supplier	RRID	Laser	Filter
Rat BD FcBlock (Purified Mouse Anti-Rat CD32)	N/A	D34-485	1/50	550273	BD Biosciences	AB_393570	N/A	N/A
Mouse Anti-Rat CD4	BUV395	OX-35	1/200	740256	BD Biosciences	AB_2740000	355 nm	379/28
Mouse Anti-Rat CD25	BUV737	OX-39	1/100	748725	BD Biosciences	AB_2873129	355 nm	740/35
Mouse Anti-Rat CD8a	BV421	OX-8	1/200	740041	BD Biosciences	AB_2739811	405 nm	450/50
Mouse Anti-Rat CD3	BV605	1F4	1/100	563949	BD Biosciences	AB_2738504	405 nm	525/50
Mouse Anti-Rat CD45	BV510	OX-1	1/150	740140	BD Biosciences	AB_2739896	405 nm	610/20
Mouse Anti-Rat CD161a	BV711	10/78	1/200	744053	BD Biosciences	AB_2741956	405 nm	710/50
Mouse Anti-Rat Itgrn AlpE2 (CD103)	BV785	OX-62	1/200	744679	BD Biosciences	AB_2742415	405 nm	780/60
Granulocyte Marker Monoclonal Antibody (His48)	FITC	HIS48	1/200	11-0570-82	eBioscience	AB_465100	488 nm	530/30
Mouse Anti-Rat RT1B	BB700	OX-6	1/200	745824	BD Biosciences	AB_2743273	488 nm	695/40
Human anti-Rat CD43	PE	REA503	1/100	130-107-684	Miltenyi Biotec	AB_2658083	561 nm	586/10
Rat Anti-Rat CD45R (B220)	PE-Cy7	HIS24	1/200	25-0460-82	eBioscience	AB_2573351	561 nm	780/60
anti-rat CD43	APC	W3/13	1/100	1614070	SONY Biotechnology	Not available	637 nm	670/14
Mouse Anti-Rat CD11b/c	R718	OX-42	1/150	752230	BD Biosciences	Not available	637 nm	730/45

Supplementary Table 2. List of fluorescence-coupled antibodies used for immunolabelling for cytometry analysis in the tumor, brain and blood

Skin toxicity

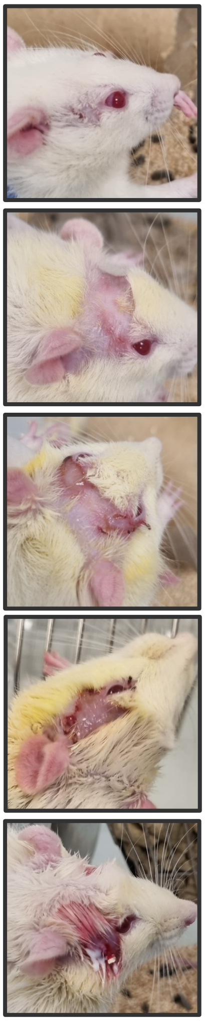

Grade 1 Dull, faint erythema, epilation

Grade 2 Bright erythema, dry desquamation

Grade 2.5 Patchy moist desquamation, moderate erythema

Grade 3 Confluent moist desquamation, pitting erythema

Grade 4 Spontaneous bleeding

Supplementary Figure 1. Illustration of the radiation dermatitis scoring.

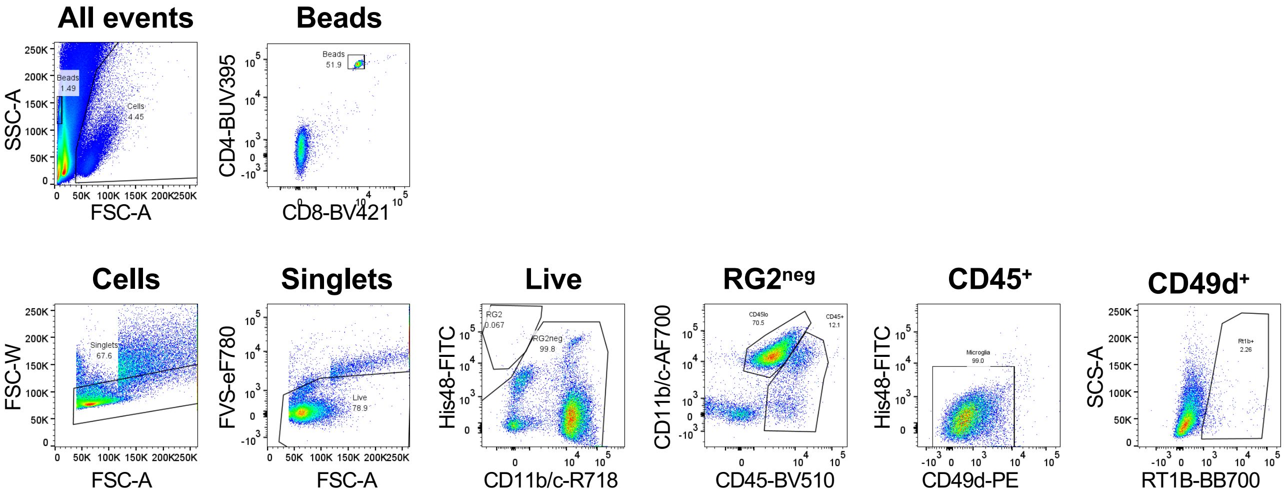

Supplementary Figure2: Gating strategy employed in the flow cytometry analysis of the brain. Representative sample of “CPT with O_2”-irradiated glioblastoma 8 days after cranial irradiation.

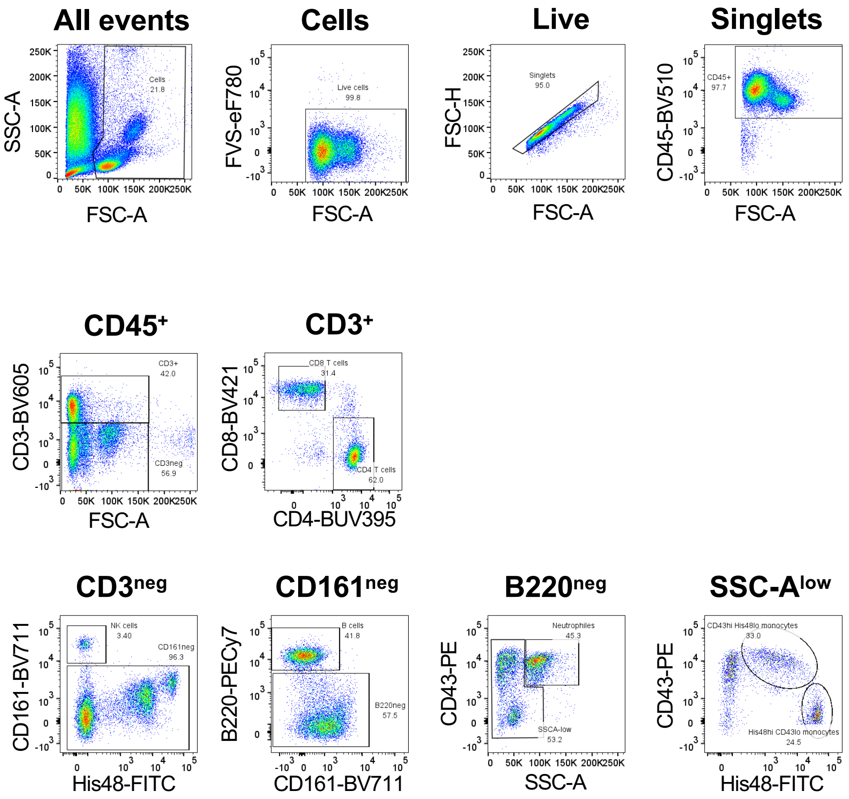

Supplementary Figure 3: Gating strategy employed in the flow cytometry analysis of the blood. Representative sample of “CPT without O_2”-irradiated glioblastoma 7 days after cranial irradiation.

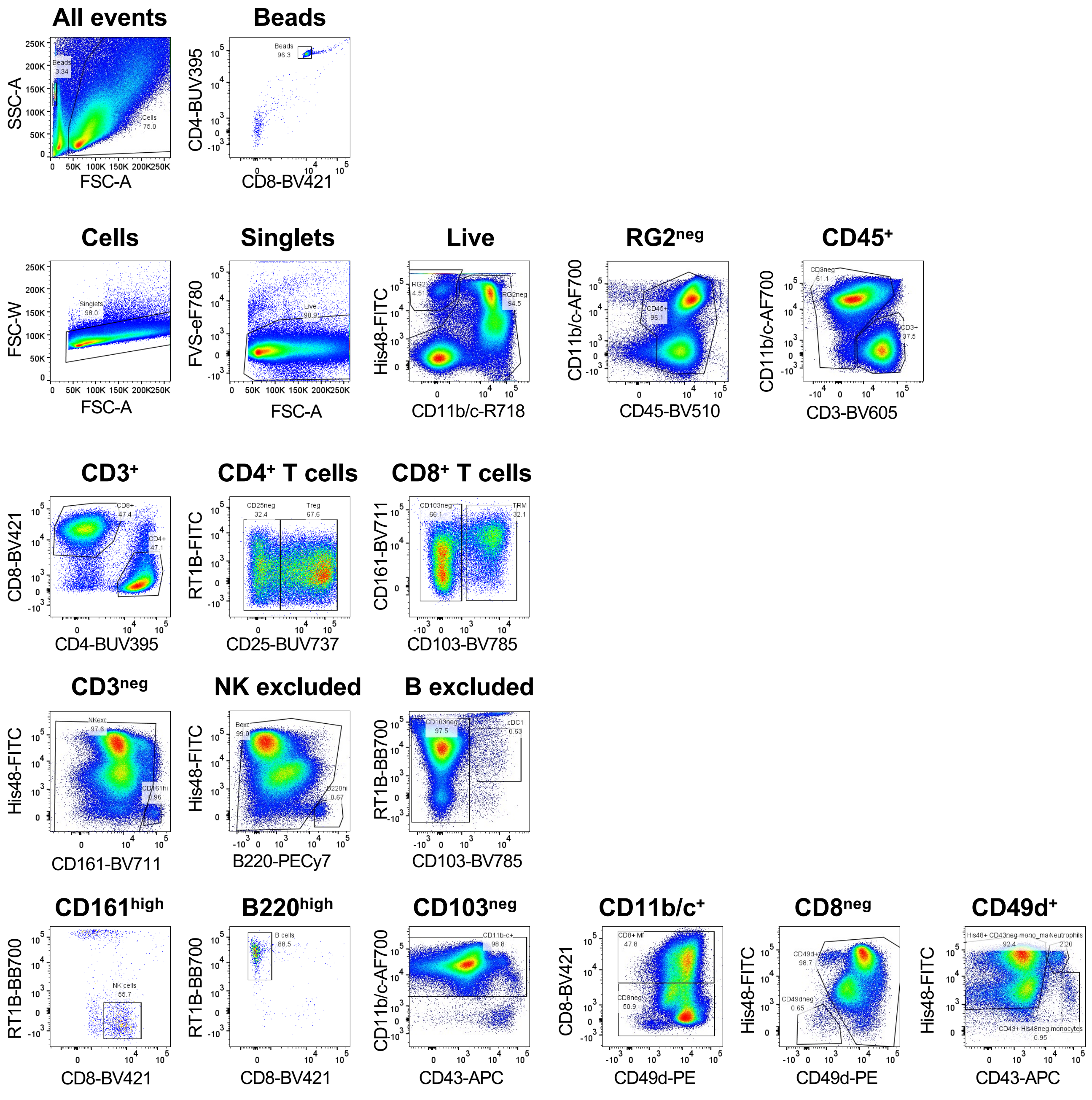

Supplementary Figure 4: Gating strategy employed in the flow cytometry analysis of the tumor. Representative sample of “CPT with O_2”-irradiated glioblastoma 8 days after cranial irradiation.

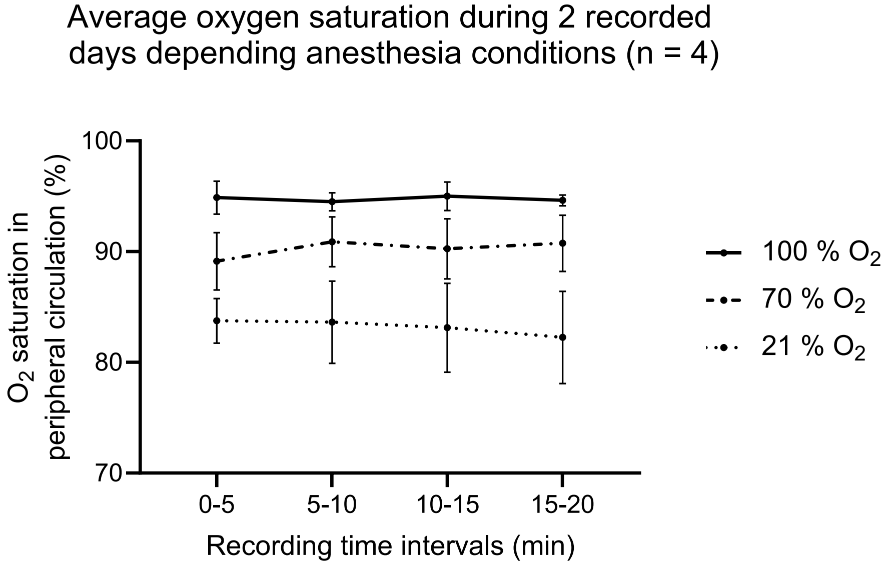

Supplementary Figure 5. Saturation of oxygen in the peripheral circulation of anesthetized rats during 20 minutes. The oxygen saturation remained stable during the recording time in the three groups. The data are presented as the mean ± standard deviation (SD).

	ANOVA table	Sum-of-squares (SS)	Degrees of freedom (DF)	Mean squares (MS)	F ratio (DFn, DFd)	P value
pFLASH with O_2 vs pFLASH without O_2	Time x O2	25.21	21	1.2	F (21, 210) = 5.922	<0.0001 (****)
	Time	222.7	21	10.6	F (21 , 210) = 52.32	<0.0001 (****)
	O2	17.77	1	17.77	F (1, 10) = 4.629	0.0569 (ns)
	Rat	38.4	10	3.84	F (10 , 210) = 18.94	0.0001 (****)
	Residual	42.56	210	0.2027		
CPT with O_2 vs CPT without O_2	Time x O2	5.905	21	0.2812	F (21, 210) = 0.9440	0.5348 (ns)
	Time	188.2	21	8.961	F (1.919, 19.19) = 30.08	<0.0001 (****)
	O2	5.47	1	5.47	F (1, 10) = 2.143	0.1740 (ns)
	Rat	25.53	10	2.553	F (10, 210) = 8.569	<0.0001 (****)
	Residual	62.56	210	0.2979		

Supplementary Table 3. Detailed ANOVA Table of Figure 1B. Detailed statistics of 2-way ANOVA comparing oxygen supplementation (upper rows for pFLASH irradiations and lower rows for conventional proton therapy, CPT). Sidak's multiple comparisons for the pFLASH groups: p-value < 0.0001 at 16 days post-irradiation (dpi), p-value < 0.0001 at 18 dpi, and p-value = 0.0002 at 21 dpi. All other p-values are non-significant. No significant differences were found by Sidak's multiple comparisons between CPT with O_2 and CPT without O_2 at any time.

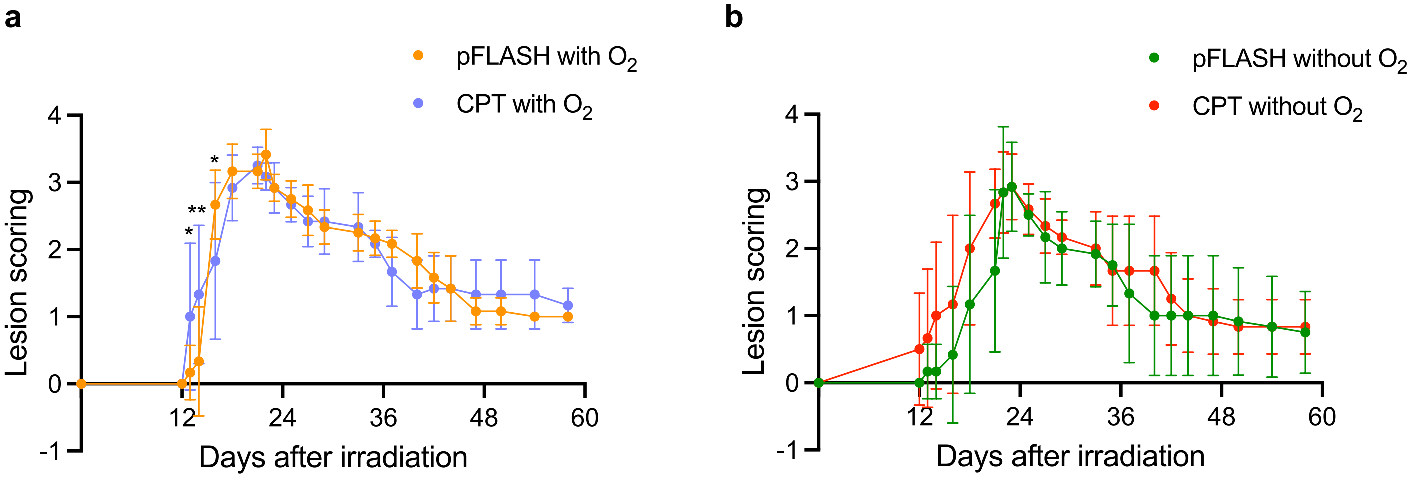

Supplementary Figure 6: Radiation dermatitis score following 25 Gy pFLASH versus conventional proton irradiations, (a) with or (b) without oxygen, n = 6 in each group. Detailed statistics (ANOVA table and multiple comparisons) are available Supplementary table 4.

	**ANOVA table**	**Sum-of-squares (SS)**	**Degrees of freedom (DF)**	**Mean squares (MS)**	**F ratio (DFn, DFd)**	**P value**
**pFLASH with O_2 vs pFLASH without O_2**	**Time x O2**	10.01	21	0.4768	F (21, 210) = 2.818	<0.0001 (****)
	**Time**	238.3	21	11.35	F (21, 210) = 67.07	<0.0001 (****)
	**O2**	0.008523	1	0.008523	F (1, 10) = 0.007459	0.9329 (ns)
	**Rat**	11.43	10	1.143	F (10, 210) = 6.753	<0.0001 (****)
	**Residual**	35.53	210	0.1692		
**CPT with O_2 vs CPT without O_2**	**Time x O2**	7.591	21	0.3615	F (21, 210) = 1.060	0.3935 (ns)
	**Time**	174.8	21	8.326	F (21, 210) = 24.42	<0.0001 (****)
	**O2**	4.909	1	4.909	F (1, 10) = 0.9690	0.3481 (ns)
	**Rat**	50.66	10	5.066	F (10, 210) = 14.86	<0.0001 (****)
	**Residual**	71.59	210	0.3409		

Supplementary Table 4. Detailed ANOVA Table of Figure S6. Detailed statistics of 2-way ANOVA comparing irradiation modes, with and without oxygen supplementation (upper rows with oxygen supplementation and lower rows in the absence of oxygen supplementation in the anesthesia gas). No significant differences were observed in at any timepoint with Sidak's multiple comparisons between pFLASH without O2 and CPT without O2. Sidak's multiple comparisons for groups with oxygen supplementation: p-value = 0.0436 at 13 dpi, p-value = 0.005 at 14 dpi, and p-value = 0.0436 at 16 dpi. All other p-values are non-significant.

Brain toxicity

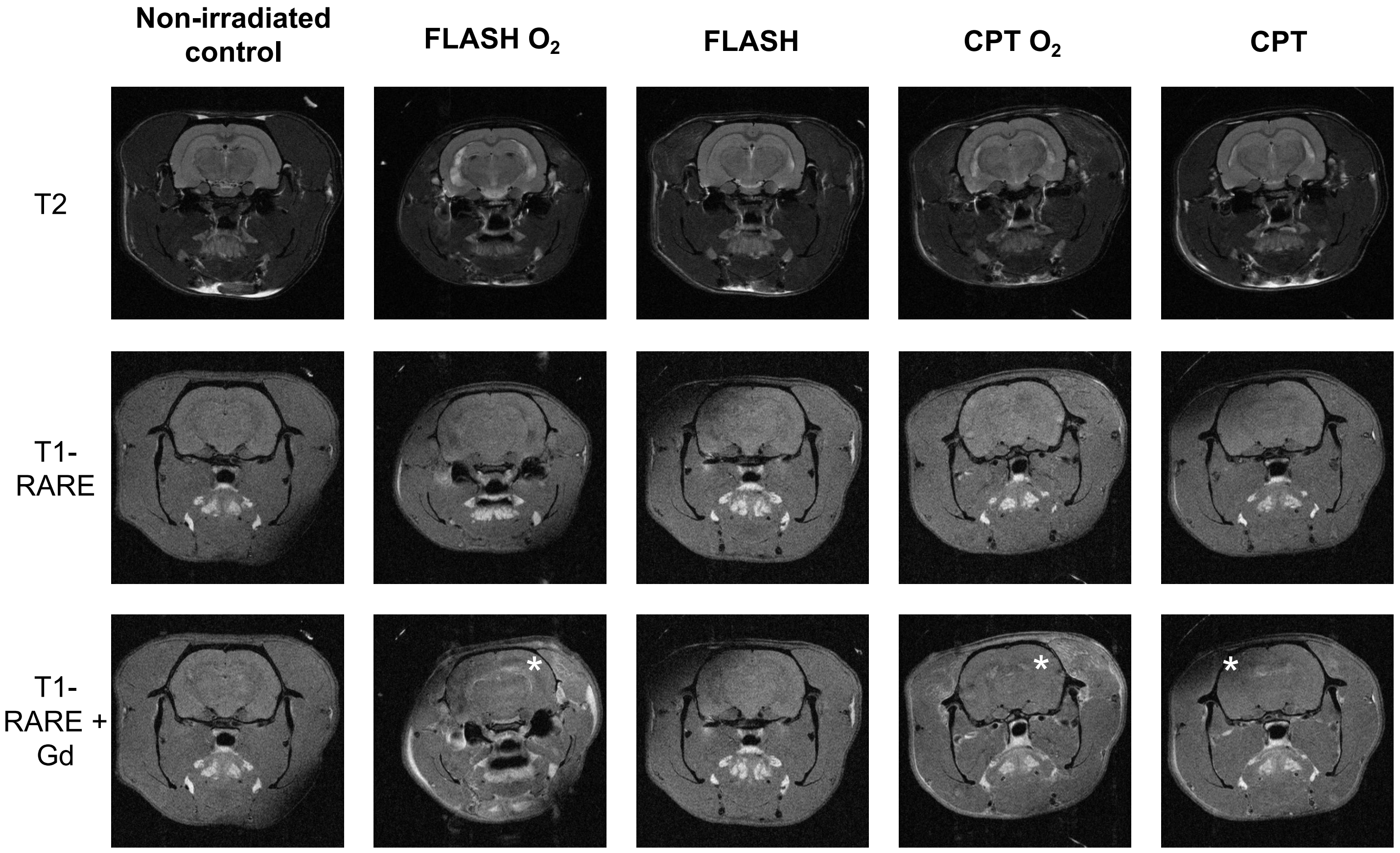

Supplementary Figure 7. **Representative MRI images of rats receiving 25 Gy, 6 months post-irradiation.** T2-weigthed, T1-weighted and T1-weighted images after gadolinium contrast reagent (Gd) was injected. Blood brain barrier leakage is observed in T1-weighted images by gadolinium-induced brightness in the brain (asterisks). Lesions are compatible with radiation necrosis in the fimbria/fornix of the hippocampus and leakage in the same region.

Brain toxicity

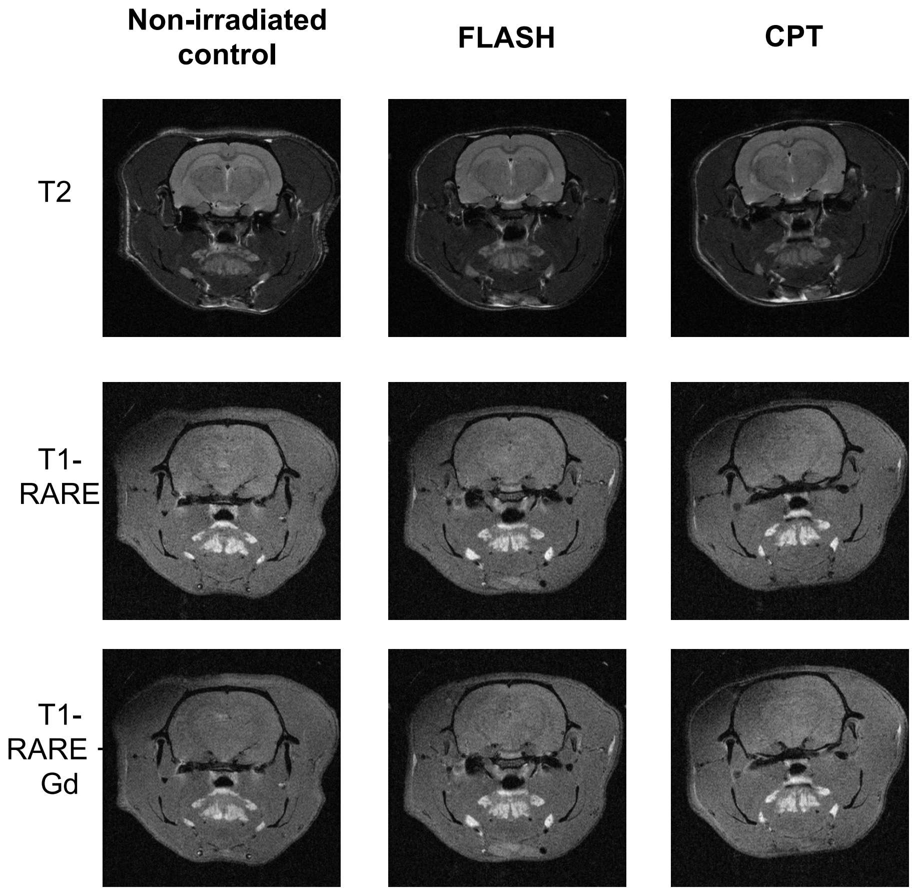

Supplementary Figure 8. **Representative MRI images of rats receiving 15 Gy, 3 months post-irradiation.** T2-weigthed and T1-weighted after gadolinium contrast reagent (Gd) was injected. No blood brain barrier leakage was observed in any of the animals.

Brain toxicity

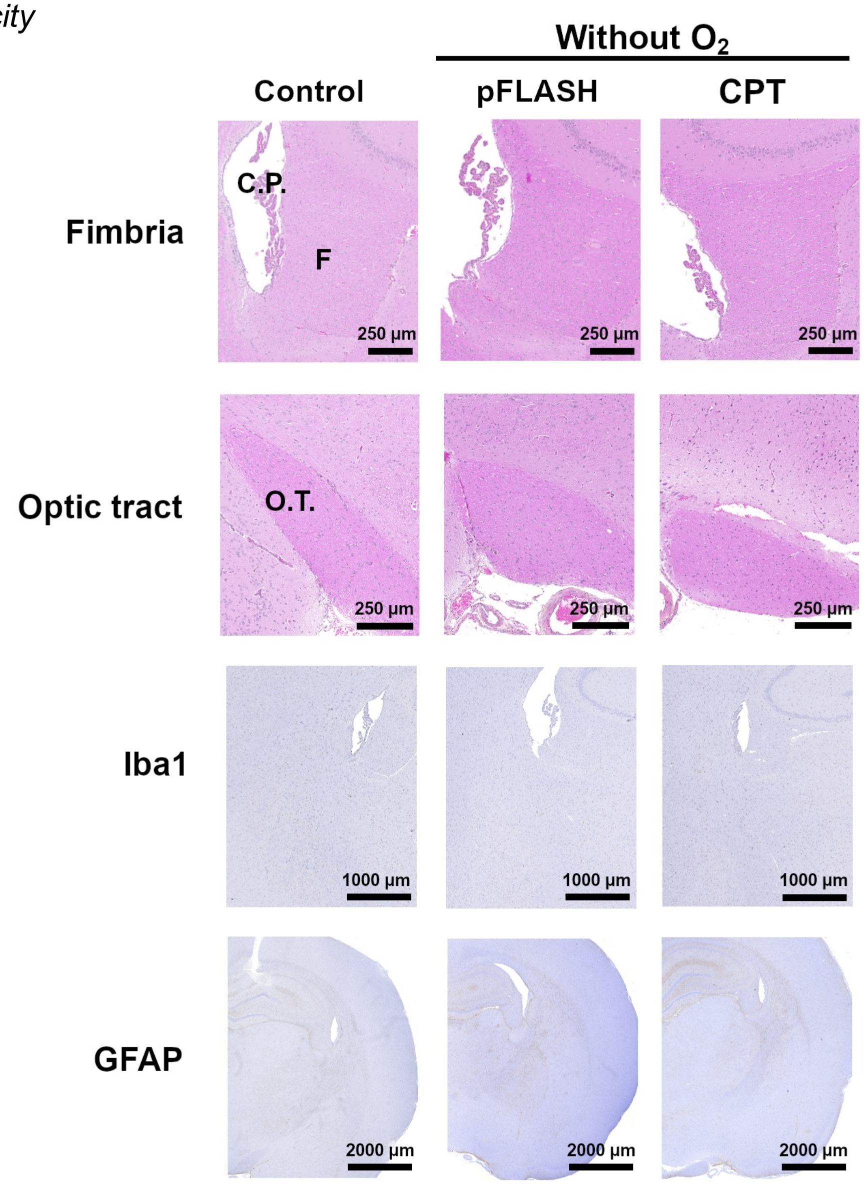

Supplementary Figure 9. Brain histology and immunohistochemistry in rats 6 months after irradiation in the 15 Gy group. No microscopic changes or modifications in Iba1 and GFAP immunolabelling patterns were observed. The first two rows present the histology of the fimbria of the hippocampus and choroid plexuses of the lateral ventricles (first row) and of the optic tracts (second raw). HE staining. Abbreviations: F: hippocampal fimbria, C.P.: choroid plexus, O.T.: optic tract. The last two rows present the immunostaining for Iba1 (third row) and GFAP (last row). Iba1 and GFAP IHC.

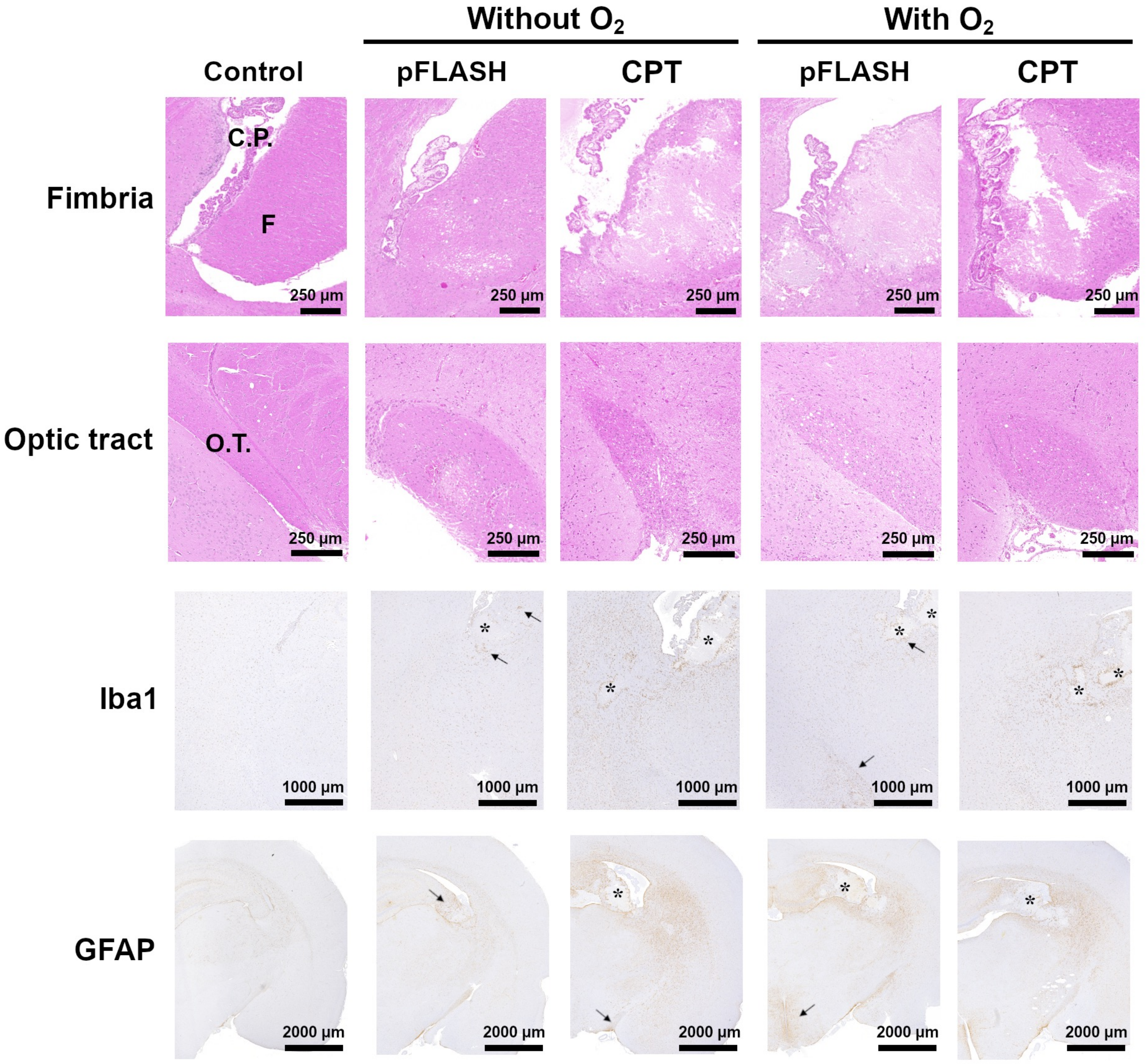

Supplementary Figure 10. Brain histopathological and immunohistochemical changes in rats 6 month after irradiation in the 25 Gy series. The first two rows present the histopathologic changes seen by hematoxylin and eosin staining (HE) in the fimbria of the hippocampus and choroid plexuses of the lateral ventricles (first row) and in the optic tracts (second row), characterized by areas of degeneration (appearing as small vacuoles or microcavities in the neuropil) to areas of necrosis (areas of pale staining). Images of a non-irradiated control rat present normal brain histology for comparison. Abbreviations: F: hippocampal fimbria, C.P.: choroid plexus, O.T. : optic tract.

The last lower rows show the immunostaining for Iba1 (third row) and GFAP (last row). An increased immunolabelling is observed in the degenerative areas and around the necrotizing areas (*) with extension to the adjacent tissues, especially for GFAP IHC. The extension of the immunolabelling for Iba1 and for GFAP was directly correlated with the severity of the histological lesions. Arrows were added to indicate areas of increased immunostaining when of small size or located to particular structures (optic tract or periventricular area of the third ventricle). Images of a non-irradiated control rat present normal brain immunostaining for comparison.

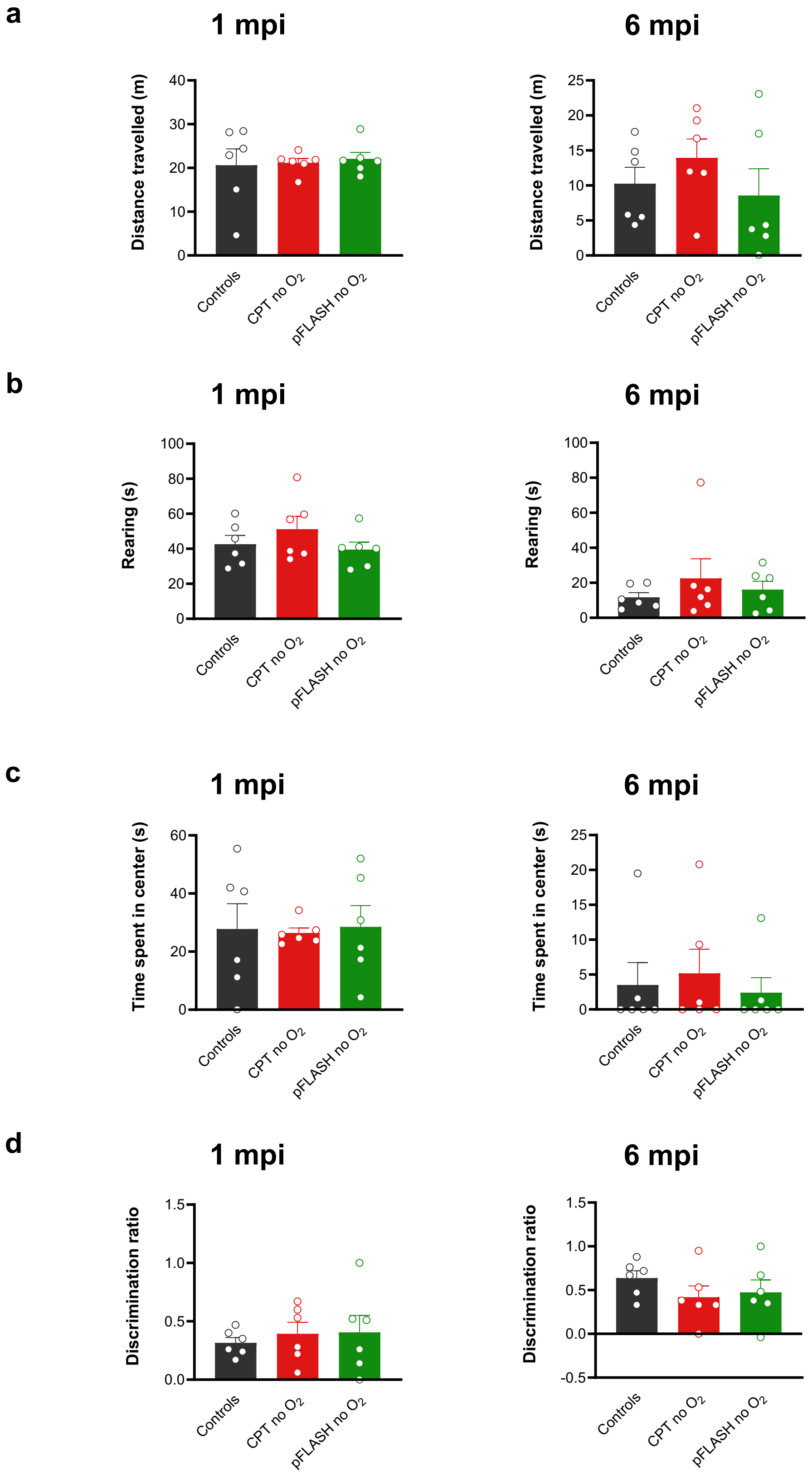

Supplementary Figure 11: Comparison of locomotor activity **(a)**, exploratory activity **(b)**, anxiety **(c)** and novel object recognition **(d)** in rats with no irradiation (Controls, dark grey) versus rats receiving 15 Gy in conventional dose rate without oxygen supplementation (CPT no O_2, dark red) and rats receiving 15 Gy in high dose rate without oxygen supplementation (pFLASH no O_2, dark green). mpi = months post-irradiation.

Supplementary Note 1. Detailed statistical analysis of the behavioral tests results presented in Figure S11:

A. Locomotor activity

1. One-month post-irradiation.

- pFlash without O_2 *vs* CPT without O_2: Independent sample T-test: t(10) = -0.5; p=0.63, Bayesian Independent sample T-test: BF10 = 0.51 in favor of the null hypothesis. *No significant difference between groups.*
- pFlash without O_2 *vs* controls: Independent sample T-test: t(10) = -0.36 ; p = 0.72, Bayesian Independent sample T-test : BF10 = 0.49 in favor of the null hypothesis. *No significant difference between groups.*
- CPT without O_2 *vs* controls: Independent sample T-test: t(10) = 0.15 ; p = 0.89, Bayesian Independent sample T-test: BF10 = 0.47 in favor of the null hypothesis. *No significant difference between groups.*

2. Six-month post-irradiation.

- pFlash without O_2 *vs* CPT without O_2: Independent sample T-test: t(10) = 1.15; p = 0.28, Bayesian Independent sample T-test: BF10 = 0.70 in favor of the null hypothesis. *No significant difference between groups.*
- pFlash without O_2 *vs* controls: Independent sample T-test: t(10) = 0.38 ; p = 0.72, Bayesian Independent sample T-test : BF10 = 0.49 in favor of the null hypothesis. *No significant difference between groups.*
- CPT without O_2 *vs* controls: Independent sample T-test: t(10) = 1.03 ; p = 0.33, Bayesian Independent sample T-test : BF10 = 0.65 in favor of the null hypothesis. *No significant difference between groups.*

The results of the analysis Jasp of **locomotor activity (ANOVA) habituation as a function of time** is shown hereafter:

- *Repeated measurements (ANOVA) for the control group:* - F(2;10) = 4.16; p = 0.049 = significative time effect, Post Hoc comparison 1 month *vs* 6 months: p = 0.049
- *Repeated measurements (ANOVA) for CPT without O_2 group:* F(2;10) = 2.93; p = 0.1 = no time effect, Post Hoc comparison: 1 month *vs* 6 months : p = 0.16
- *Repeated measurements (ANOVA) for pFLASH without O_2 group:* F(2;10) = 9.39; p = 0.005 = significative time effect, Post Hoc comparison 1 month *vs* 6 months: p = 0.005

B. Exploratory activity

1. One-month post-irradiation: exploratory activity.

- pFlash without O_2 *vs* CPT without O_2: Independent sample T-test: t(10) = 1.38; p = 0.2, Bayesian Independent sample T-test: BF10 = 0.83 in favor of the null hypothesis. *No significant difference between groups.*
- pFlash without O_2 *vs* controls: Independent sample T-test: t(10) = 0.47 ; p = 0.65, Bayesian Independent sample T-test: BF10 = 0.5 in favor of the null hypothesis. *No significant difference between groups.*
- CPT without O_2 *vs* controls: Independent sample T-test: t(10) = 0.97; p = 0.36, Bayesian independent sample T-test : BF10 = 0.63 in favor of the null hypothesis. *No significant difference between groups.*

2. Six-month post-irradiation: exploratory activity

- pFlash without O_2 *vs* CPT without O_2: Independent sample T-test: t(10) = 0.53 ; p = 0.61, Bayesian Independent sample T-test: BF10 = 0.51 in favor of the null hypothesis. *No significant difference between groups.*
- pFlash without O_2 *vs* controls: Independent sample T-test: t(10) = -0.8 ; p = 0.44, Bayesian Independent sample T-test : BF10 = 0.57 in favor of the null hypothesis. *No significant difference between groups.*
- CPT without O_2 *vs* controls: Independent sample T-test: t(10) = 0.94 ; p = 0.37, Bayesian Independent sample T-test : BF10 = 0.62 in favor of the null hypothesis. *No significant difference between groups.*

The results of the analysis Jasp of **exploratory activity (ANOVA) habituation as a function of time** are shown hereafter:

- *Repeated measurements (ANOVA) for the control group:* F(2;10) = 14,04 ; p = 0.001 = significative time effect, Post Hoc comparison: 1 month *vs* 6 months : p = 0,001
- *Repeated measurements (ANOVA) for CPT without O_2 group:* F(2;10) = 12.19; p = 0.002 = significative time effect, Post Hoc comparison: 1 month *vs* 6 months: p = 0.003
- *Repeated measurements (ANOVA) for pFLASH without O_2 group:* F(2;10) = 12.09 ; p = 0.002 = significative time effect, Post Hoc comparison : 1 month *vs* 6 months: p = 0.002

C. Anxiety

1. One-month post-irradiation

- pFlash without O_2 *vs* CPT without O2: Independent sample T-test: t(10)= -0,28 ; p = 0.79, Bayesian Independent sample T-test : BF10 = 0.48 in favor of the null hypothesis. *No significant difference between groups.*
- pFlash without O_2 *vs* controls: Independent sample T-test: t(10) = -0,07; p = 0.95, Bayesian Independent sample T-test : BF10 = 0.47 in favor of the null hypothesis. *No significant difference between groups.*
- CPT without O_2 *vs* controls: Independent sample T-test: t(10)= -0.15; p = 0.88, Bayesian Independent sample T-test : BF10 = 0.47 in favor of the null hypothesis. *No significant difference between groups.*

2. Six-month post-irradiation

- pFlash without O_2 *vs* CPT without O_2 : Independent sample T-test: t(10) = 0.68 ; p = 0.51, Bayesian Independent sample T-test: BF10 = 0.54 in favor of the null hypothesis. *No significant difference between groups.*
- pFlash without O_2 *vs* controls: Independent sample T-test: t(10) = 0.29; p = 0.78, Bayesian Independent sample T-test : BF10 = 0.48 in favor of the null hypothesis. *No significant difference between groups.*
- CPT without O_2 *vs* controls: Independent sample T-test: t(10) = 0.35; p = 0.73, Bayesian Independent sample T-test : BF10 = 0.49 in favor of the null hypothesis. *No significant difference between groups.*

The results of the analysis Jasp of **time spent in the center of the open area test (ANOVA) as a function of time** are shown hereafter:

- *Repeated measurements (ANOVA) for the control group:* F(2;10) = 1.29; p = 0.002 = significative time effect - Post Hoc comparison: 1 month *vs* 6 months: p = 0,004
- *Repeated measurements (ANOVA) for CPT without O_2 group*: F(2;10) = 16.59; p < 0.001 = significative time effect - Post Hoc comparison : 1 month *vs* 6 months: p < 0.001
- *Repeated measurements (ANOVA) for pFLASH without O_2 group*: F(2;10) = 10.94; p = 0.003 = significative time effect, Post Hoc comparison : 1 month *vs* 6 months : p = 0.003

D. Novel object recognition

1. One-month post-irradiation

- pFlash without O_2 *vs* CPT without O_2: Independent sample T-test: t(10) = -0.06; p = 0.96, Bayesian Independent sample T-test : BF10 = 0.47 in favor of the null hypothesis. *No significant difference between groups.*
- pFlash without O_2 *vs* controls: Independent sample T-test: t(10)= -0.58; p = 0.57, Bayesian Independent sample T-test: BF10 = 0.52 in favor of the null hypothesis. *No significant difference between groups.*
- CPT without O_2 *vs* controls: Independent sample T-test: t(10) = -0.72; p = 0.49, Bayesian Independent sample T-test: BF10 = 0.55 in favor of the null hypothesis. *No significant difference between groups.*

Compared to 0: >0

- Controls: One sample T-Test: t(5) = 6.92; p < 0.001, Bayesian one sample T-Test: BF+0 = 82.05 = in favor of the alternative hypothesis = superior to 0
- CPT without O_2: One sample T-Test: t(5) = 4.02; p = 0.005, Bayesian one sample T-Test : BF+0 = 13.57 = in favor of the alternative hypothesis = superior to 0
- pFLASH without O_2: One sample T-Test: t(5)=2,8 ; p=0,019, Bayesian one sample T-Test : BF+0 = 4,97 = in favor of the alternative hypothesis = superior to 0

2. Six-month post-irradiation

- pFlash without O_2 *vs* CPT without O_2: Independent sample T-test: t(10) = -0.28; p = 0.79, Bayesian Independent sample T-test : BF10 = 0.48 in favor of the null hypothesis. *No significant difference between groups.*
- pFlash without O_2 *vs* controls: Independent sample T-test: t(10) = 0.99; p = 0.34, Bayesian Independent sample T-test: BF10 = 0.64 in favor of the null hypothesis. *No significant difference between groups.*
- CPT without O_2 *vs* controls: Independent sample T-test: t(10)= 1,42 ; p=0,19, Bayesian Independent sample T-test : BF10=0,86 in favor of the null hypothesis. *No significant difference between groups.*

Compared to 0: >0

- Controls: One sample T-Test: t(5) = 7.75; p < 0.001, Bayesian one sample T-Test: BF+0 = 123.09 = in favor of the alternative hypothesis = superior to 0
- CPT without O_2: One sample T-Test: t(5) = 3.31; p = 0.01, Bayesian one sample T-Test: BF+0 = 7.67 = in favor of the alternative hypothesis = superior to 0
- pFLASH without O_2: One sample T-Test: t(5) = 3.37; p = 0.01, Bayesian one sample T-Test: BF+0 = 8.1 = in favor of the alternative hypothesis = superior to 0

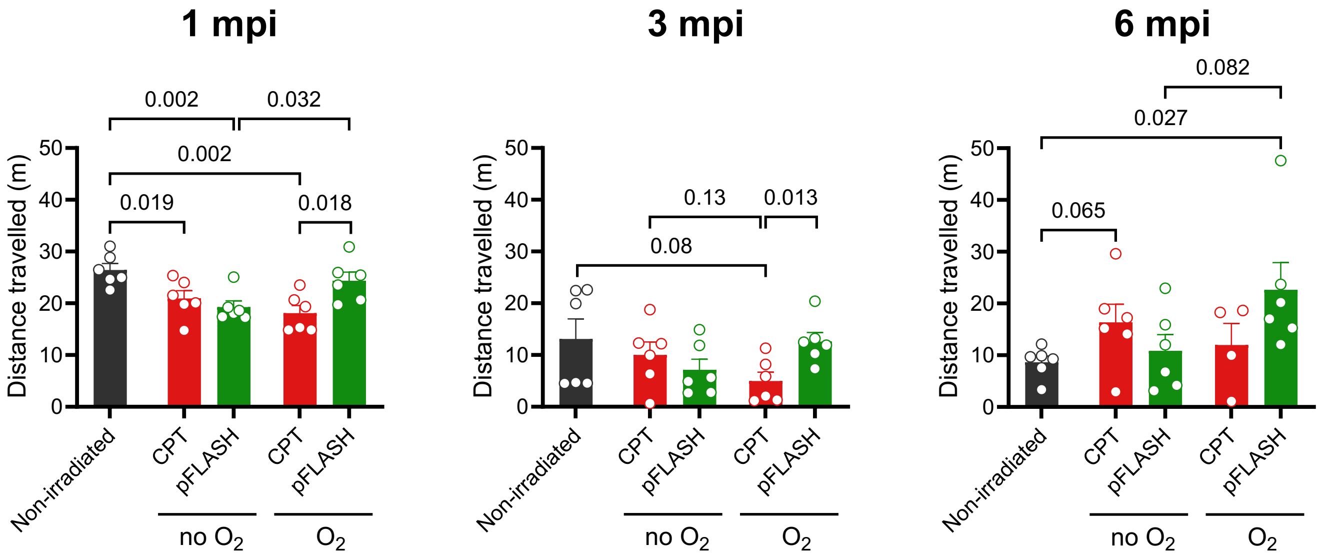

Supplementary Figure 12. Comparison of locomotor activity in 5 minutes open field test in rats with no irradiation (Controls, dark grey) versus rats receiving 25 Gy in conventional dose rate (CPT, dark red) or in high dose rate (pFLASH, dark green) with or without oxygen supplementation (O_2) during anesthesia.

	Locomotor activity (decreased activity)			
	**Global decrease**	**1 vs 3 mpi**	**1 vs 6 mpi**	**Memorization of context**
**Not irradiated controls**	F(2;10) = 20.7, p < 0.001 (***)	p = 0.002 (**)	p < 0.001 (***)	**Not altered**
**CPT without O_2**	F(2;10) = 5.3, p = 0.03 (*)	p = 0.03 (*)	p = 0.2 **(ns)**	**Altered at 6 mpi**
**CPT O_2**	F(2;10) = 9.3, p = 0.015 (*)	p = 0.016 (*)	p = 0.15 **(ns)**	**Altered at 6 mpi**
**pFLASH without O_2**	F(2;10) = 13.4, p = 0.001 (***)	p = 0.001 (***)	p = 0.01 (**)	**Not altered**
**pFLASH O_2**	F(2;10) = 3.15, p = 0.09 **(ns)**	p = 0.13 **(ns)**	p = 0.7 **(ns)**	**Altered from 3 mpi**

Supplementary Table 5. Analysis Jasp of locomotor activity (ANOVA) habituation as a function of time. *Repeated measurements* over the months post-irradiation for each group. Only non-irradiated controls and pFLASH with O_2 animals had a normal decreased locomotor activity over the months post-irradiation, meaning a not altered memorization of context.

Supplementary Note 2. Detailed statistical analysis of the behavioral tests results presented in Figure S12:

1. One-month post-irradiation.

- pFlash without O_2 *vs* CPT without O_2: Independent sample T-test: $t(10) = 0.86$; $p=0.41$, Bayesian Independent sample T-test: BF10 = 0.59 in favor of the null hypothesis. *No significant difference between groups.*
- pFlash without O_2 *vs* controls: Independent sample T-test: $t(10) = 4.13$; $p = 0.002$, Bayesian Independent sample T-test : BF10 = 16.32 in favor of the alternative hypothesis. S*ignificant difference between groups.*
- CPT without O_2 *vs* controls: Independent sample T-test: $t(10) = -2.8$; $p = 0.02$, Bayesian Independent sample T-test: BF10 = 3.36 in favor of the alternative hypothesis. *Significant difference between groups.*

- pFlash with O_2 *vs* CPT with O_2: Independent sample T-test: $t(10) = -2.82$; $p=0.018$, Bayesian Independent sample T-test: BF10 = 3.5 in favor of the alternative hypothesis. *Significant difference between groups.*
- pFlash with O_2 *vs* controls: Independent sample T-test: $t(10) = 0.99$; $p = 0.35$, Bayesian Independent sample T-test : BF10 = 0.64 in favor of the null hypothesis. *No significant difference between groups.*
- CPT with O_2 *vs* controls: Independent sample T-test: $t(10) = -4.3$; $p = 0.002$, Bayesian Independent sample T-test: BF10 = 19.72 in favor of the alternative hypothesis. *Significant difference between groups.*

- CPT without O_2 *vs* CPT with O_2: Independent sample T-test: $t(10) = 1.34$; $p=0.2$, Bayesian Independent sample T-test: BF10 = 0.8 in favor of the null hypothesis. *No significant difference between groups.*
- pFlash without O_2 *vs pFlash* with O_2 : Independent sample T-test: $t(10) = -2.5$; $p = 0.03$, Bayesian Independent sample T-test : BF10 = 2.42 in favor of the alternative hypothesis. S*ignificant difference between groups.*

2. Three-month post-irradiation.

- pFlash without O_2 *vs* CPT without O_2: Independent sample T-test: $t(10) = -2.4$; $p=0.4$, Bayesian Independent sample T-test: BF10 = 0.6 in favor of the null hypothesis. *No significant difference between groups.*
- pFlash without O_2 *vs* controls: Independent sample T-test: $t(10) = 1.38$; $p = 0.2$, Bayesian Independent sample T-test : BF10 = 0.83 in favor of the null hypothesis. *No significant difference between groups.*
- CPT without O_2 *vs* controls: Independent sample T-test: $t(10) = -0.68$; $p = 0.52$, Bayesian Independent sample T-test: BF10 = 0.54 in favor of the null hypothesis. *No significant difference between groups.*

- pFlash with O_2 *vs* CPT with O_2: Independent sample T-test: $t(10) = -3.08$; $p=0.012$, Bayesian Independent sample T-test: BF10 = 4.73 in favor of the alternative hypothesis. *Significant difference between groups.*
- pFlash with O_2 *vs* controls: Independent sample T-test: $t(10) = 0.13$; $p = 0.9$, Bayesian Independent sample T-test : BF10 = 0.47 in favor of the null hypothesis. *No significant difference between groups.*
- CPT with O_2 *vs* controls: Independent sample T-test: $t(10) = -1.94$; $p = 0.08$, Bayesian Independent sample T-test: BF10 = 1.36 in favor of the alternative hypothesis. *Significant difference between groups.*

- CPT without O_2 *vs* CPT with O_2: Independent sample T-test: $t(10) = 1.66$; $p=0.13$, Bayesian Independent sample T-test: BF10 = 1.05 in favor of the alternative hypothesis. *Significant difference between groups.*
- pFlash without O_2 *vs pFlash* with O_2 : Independent sample T-test: $t(10) = -2$; $p = 0.07$, Bayesian Independent sample T-test : BF10 = 1.45 in favor of the alternative hypothesis. S*ignificant difference between groups.*

3. Six-month post-irradiation.

- pFlash without O_2 *vs* CPT without O_2: Independent sample T-test: t(10) = 1.17; p=0.27, Bayesian Independent sample T-test: BF10 = 0.71 in favor of the null hypothesis. *No significant difference between groups.*
- pFlash without O_2 *vs* controls: Independent sample T-test: t(10) = -0.65 ; p = 0.53, Bayesian Independent sample T-test : BF10 = 0.54 in favor of the null hypothesis. *No significant difference between groups.*
- CPT without O_2 *vs* controls: Independent sample T-test: t(10) = 2.07 ; p = 0.065, Bayesian Independent sample T-test: BF10 = 1.55 in favor of the alternative hypothesis. *Significant difference between groups.*

- pFlash with O_2 *vs* CPT with O_2: Independent sample T-test: t(8) = -1.45; p=0.18, Bayesian Independent sample T-test: BF10 = 0.9 in favor of the null hypothesis. *No significant difference between groups.*
- pFlash with O_2 *vs* controls: Independent sample T-test: t(10) = -2.6 ; p = 0.027, Bayesian Independent sample T-test : BF10 = 2.7 in favor of the alternative hypothesis. *Significant difference between groups.*
- CPT with O_2 *vs* controls: Independent sample T-test: t(8) = 0.93 ; p = 0.38, Bayesian Independent sample T-test: BF10 = 0.64 in favor of the null hypothesis. *No significant difference between groups.*

- CPT without O_2 *vs* CPT with O_2: Independent sample T-test: t(8) = 0.8; p=0.45, Bayesian Independent sample T-test: BF10 = 0.6 in favor of the null hypothesis. *No significant difference between groups.*
- pFlash without O_2 *vs pFlash* with O_2 : Independent sample T-test: t(10) = -1.93 ; p = 0.082, Bayesian Independent sample T-test : BF10 = 1.35 in favor of the alternative hypothesis. S*ignificant difference between groups.*

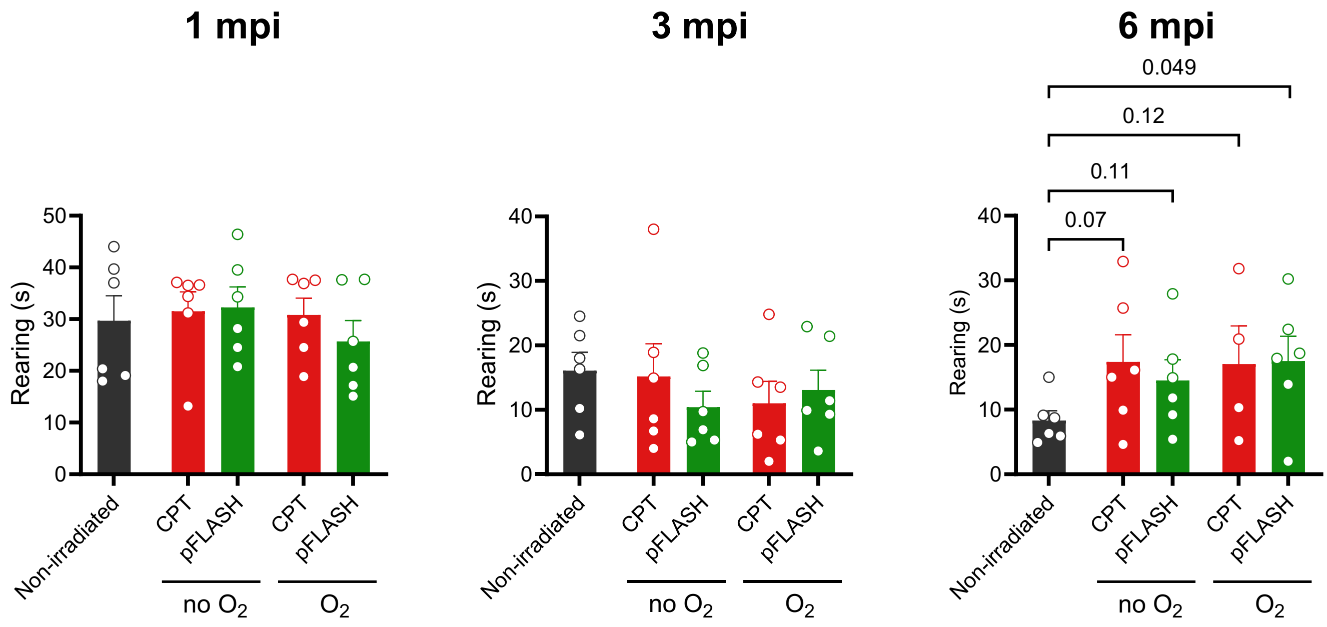

Supplementary Figure 13. Comparison of exploratory activity in rats with no irradiation (Controls, dark grey) versus rats receiving 25 Gy in conventional dose rate (CPT, dark red) or in high dose rate (pFLASH, dark green) with or without oxygen supplementation (O_2) during anesthesia.

Exploratory activity (decreased activity)

	**Global decrease**	**1 vs 3 mpi**	**1 vs 6 mpi**	**Memorization of context**
**Not irradiated controls**	$F(2;10) = 19.9$, $p < 0.001$ (***)	$p = 0.005$ (**)	$p < 0.001$ (***)	**Not altered**
**CPT without O_2**	$F(2;10) = 3.9$, $p = 0.06$ **(ns)**	$p = 0.08$ **(ns)**	$p = 0.1$ **(ns)**	**Altered from 3 mpi**
**CPT O_2**	$F(2;10) = 5.2$, $p = 0.05$ (*)	$p = 0.05$ (*)	$p = 0.2$ **(ns)**	**Altered at 6 mpi**
**pFLASH without O_2**	$F(2;10) = 21.7$, $p < 0.001$ (***)	$p < 0.001$ (***)	$p = 0.001$ (***)	**Not altered**
**pFLASH O_2**	$F(2;10) = 5.1$, $p = 0.03$ (*)	$p = 0.03$ (*)	$p = 0.14$ **(ns)**	**Altered at 6 mpi**

Supplementary Table 6. Analysis Jasp of exploratory activity (ANOVA) for habituation as a function of time. *Repeated measurements* over the months post-irradiation for each group. Only not irradiated controls and pFLASH with O_2 animals had a normal decreased exploratory activity over the months post-irradiation, meaning a not altered memorization of context.

Supplementary Note 3. Detailed statistical analysis of the behavioral tests results presented in Figure S13:

1. One-month post-irradiation.

- pFlash without O_2 *vs* CPT without O_2: Independent sample T-test: t(10) = 0.14; p=0.89, Bayesian Independent sample T-test: BF10 = 0.47 in favor of the null hypothesis. *No significant difference between groups.*
- pFlash without O_2 *vs* controls: Independent sample T-test: t(10) = -0.42 ; p = 0.69, Bayesian Independent sample T-test : BF10 = 0.49 in favor of the null hypothesis. *No significant difference between groups.*
- CPT without O_2 *vs* controls: Independent sample T-test: t(10) = 0.3 ; p = 0.77, Bayesian Independent sample T-test: BF10 = 0.48 in favor of the null hypothesis. *No significant difference between groups.*

- pFlash with O_2 *vs* CPT with O_2: Independent sample T-test: t(10) = 0.99; p=0.34, Bayesian Independent sample T-test: BF10 = 0.64 in favor of the null hypothesis. *No significant difference between groups.*
- pFlash with O_2 *vs* controls: Independent sample T-test: t(10) = -0.64 ; p = 0.54, Bayesian Independent sample T-test : BF10 = 0.53 in favor of the null hypothesis. *No significant difference between groups.*
- CPT with O_2 *vs* controls: Independent sample T-test: t(10) = 0.19 ; p = 0.85, Bayesian Independent sample T-test: BF10 = 0.47 in favor of the null hypothesis. *No significant difference between groups.*

- CPT without O_2 *vs* CPT with O_2: Independent sample T-test: t(10) = 0.14; p=0.89, Bayesian Independent sample T-test: BF10 = 0.47 in favor of the null hypothesis. *No significant difference between groups.*
- pFlash without O_2 *vs pFlash* with O_2 : Independent sample T-test: t(10) = 1.17 ; p = 0.27, Bayesian Independent sample T-test : BF10 = 0.71 in favor of the null hypothesis. *No significant difference between groups.*

2. Three-month post-irradiation.

- pFlash without O_2 *vs* CPT without O_2: Independent sample T-test: t(10) = 0.84; p=0.42, Bayesian Independent sample T-test: BF10 = 0.58 in favor of the null hypothesis. *No significant difference between groups.*
- pFlash without O_2 *vs* controls: Independent sample T-test: t(10) = 1.52 ; p = 0.16, Bayesian Independent sample T-test : BF10 = 0.9 in favor of the null hypothesis. *No significant difference between groups.*
- CPT without O_2 *vs* controls: Independent sample T-test: t(10) = -0.15 ; p = 0.88, Bayesian Independent sample T-test: BF10 = 0.47 in favor of the null hypothesis. *No significant difference between groups.*

- pFlash with O_2 *vs* CPT with O_2: Independent sample T-test: t(10) = 0.45; p=0.66, Bayesian Independent sample T-test: BF10 = 0.5 in favor of the null hypothesis. *No significant difference between groups.*
- pFlash with O_2 *vs* controls: Independent sample T-test: t(10) = 0.72 ; p = 0.49, Bayesian Independent sample T-test : BF10 = 0.55 in favor of the null hypothesis. *No significant difference between groups.*
- CPT with O_2 *vs* controls: Independent sample T-test: t(10) = -0.15 ; p = 0.28, Bayesian Independent sample T-test: BF10 = 0.71 in favor of the null hypothesis. *No significant difference between groups.*

- CPT without O_2 *vs* CPT with O_2: Independent sample T-test: t(10) = 0.68; p=0.5, Bayesian Independent sample T-test: BF10 = 0.54 in favor of the null hypothesis. *No significant difference between groups.*
- pFlash without O_2 *vs pFlash* with O_2 : Independent sample T-test: t(10) = -0.67 ; p = 0.52, Bayesian Independent sample T-test : BF10 = 0.54 in favor of the null hypothesis. *No significant difference between groups.*

3. Six-month post-irradiation.

- pFlash without O_2 *vs* CPT without O_2: Independent sample T-test: t(10) = 0.54; p=0.6, Bayesian Independent sample T-test: BF10 = 0.51 in favor of the null hypothesis. *No significant difference between groups.*
- pFlash without O_2 *vs* controls: Independent sample T-test: t(10) = -1.75 ; p = 0.11, Bayesian Independent sample T-test : BF10 = 1.13 in favor of the alternative hypothesis. *Significant difference between groups.*
- CPT without O_2 *vs* controls: Independent sample T-test: t(10) = 2.02 ; p = 0.07, Bayesian Independent sample T-test: BF10 = 1.47 in favor of the alternative hypothesis. *Significant difference between groups.*

- pFlash with O_2 *vs* CPT with O_2: Independent sample T-test: t(8) = -0.7; p=0.95, Bayesian Independent sample T-test: BF10 = 0.5 in favor of the null hypothesis. *No significant difference between groups.*
- pFlash with O_2 *vs* controls: Independent sample T-test: t(10) = -2.24 ; p = 0.049, Bayesian Independent sample T-test : BF10 = 1.84 in favor of the alternative hypothesis. *Significant difference between groups.*
- CPT with O_2 *vs* controls: Independent sample T-test: t(8) = 1.74 ; p = 0.12, Bayesian Independent sample T-test: BF10 = 1.13 in favor of the alternative hypothesis. *Significant difference between groups.*

- CPT without O_2 *vs* CPT with O_2: Independent sample T-test: t(8) = 0.045 ; p=0.97, Bayesian Independent sample T-test: BF10 = 0.5 in favor of the null hypothesis. *No significant difference between groups.*
- pFlash without O_2 *vs pFlash* with O_2 : Independent sample T-test: t(10) = -0.6 ; p = 0.56, Bayesian Independent sample T-test : BF10 = 0.53 in favor of the null hypothesis. *No significant difference between groups.*

Time spent in the center (decreased activity)				
	**Global decrease**	**1 vs 3 mpi**	**1 vs 6 mpi**	**Memorization of context**
**Not irradiated controls**	F(2;10) = 6.5, p = 0.016 (*)	p = 0.05 (*)	p = 0.02 (*)	**Not altered**
**CPT without O_2**	F(2;10)=8.5, p=0.007 (**)	p = 0.008 (**)	p = 0.025 (*)	**Not altered**
**CPT O_2**	F(2;6) = 2.1, p = 0.2 (ns)	p = 0.46 **(ns)**	p = 0.5 **(ns)**	**Altered from 3 mpi**
**pFLASH without O_2**	F(2;10) = 12.4, p = 0.002 (**)	p = 0.004 (**)	p = 0.004 (**)	**Not altered**
**pFLASH O_2**	F(2;10) = 6.13, p = 0.018 (*)	p = 0.017 (*)	p = 0.2 **(ns)**	**Altered at 6 mpi**

Supplementary Table 7. Analysis Jasp of time spent in the center (ANOVA) for habituation as a function of time. Repeated measurements over the months post-irradiation for each group. Only not irradiated controls, CPT without O_2 and pFLASH without O_2 animals had a normal decreased anxiety measurement over the months post-irradiation, meaning a not altered memorization of context.

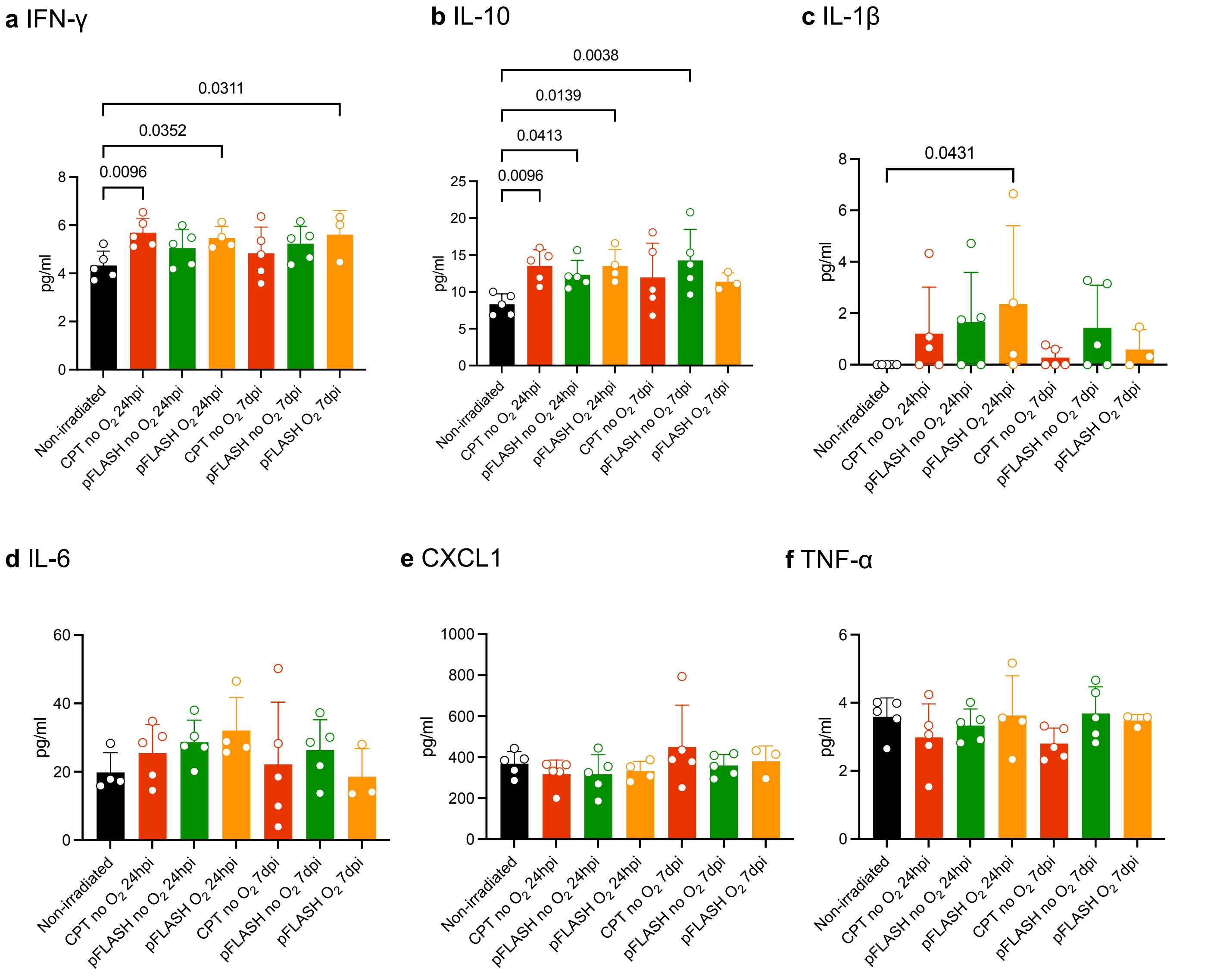

Supplementary Figure 14. Concentrations of systemic pro-inflammatory cytokines, at 24 hours post irradiation (24hpi) and 7 days post irradiation (7dpi) after pFLASH or conventional proton irradiations for (a) IFN-γ, (b) IL-10, (c) IL-1β, (d) IL-6, (e) CXCL1 and, (f) TNF-α. hpi = hours post-irradiation, dpi = days post-irradiation. The data are presented as the mean ± standard deviation (SD).

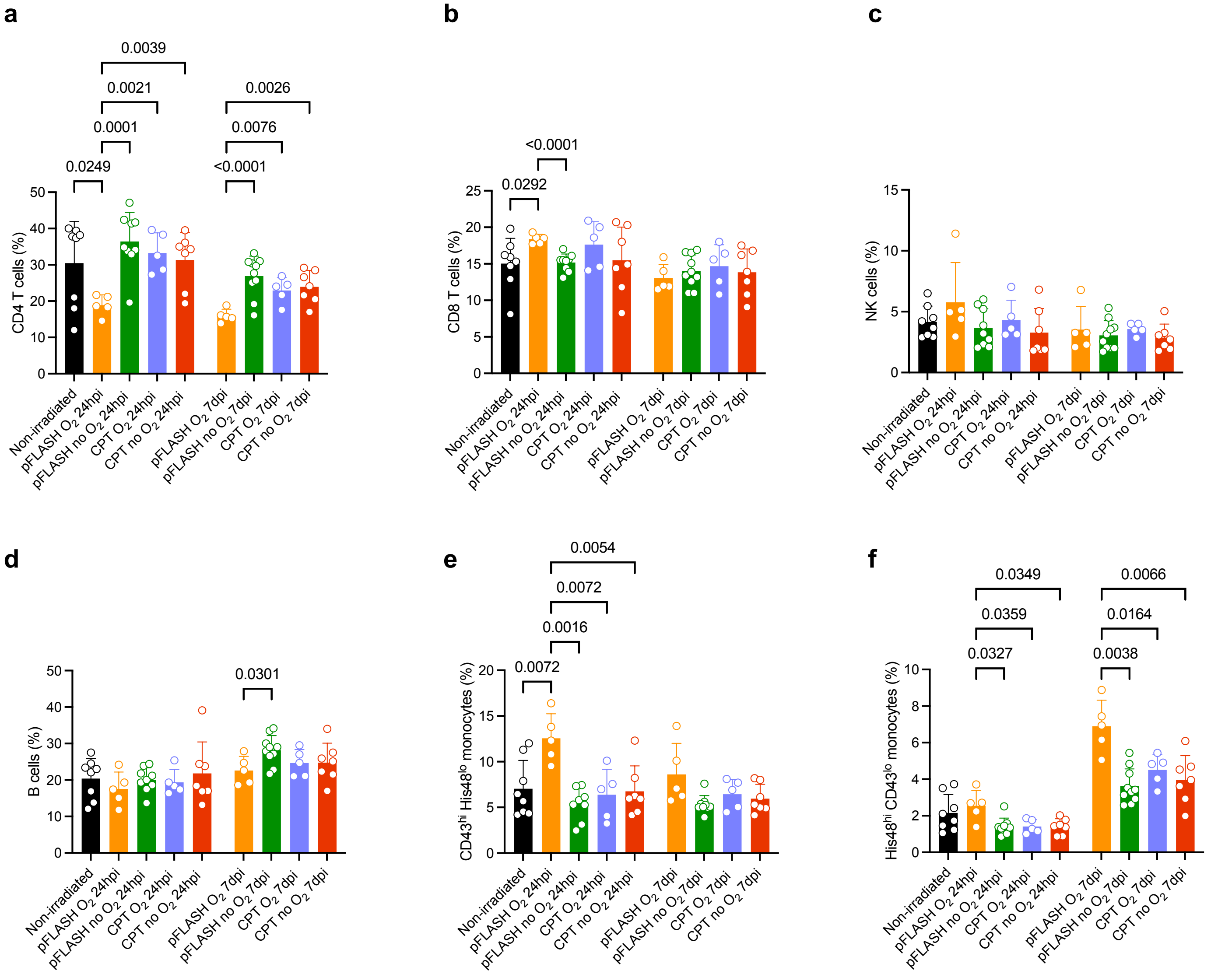

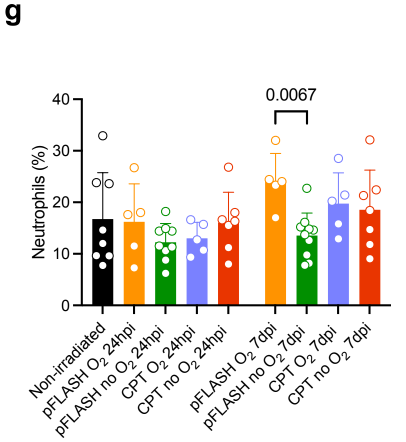

h

	Two-way ANOVA	Source of variation (P value)		
		Interaction	Oxygen	Dose rate
24 hours post irradiation (hpi)	CD4 T cells	0.0018 (**)	0.0094 (**)	0.1028 (ns)
	CD8 T cells	0.6489 (ns)	0.0281 (*)	0.8541 (ns)
	NK cells	0.5319 (ns)	0.0793 (ns)	0.2829 (ns)
	B cells	0.9972 (ns)	0.2779 (ns)	0.4294 (ns)
	His48^{+} monocytes	0.0143 (*)	0.0136 (*)	0.0175 (*)
	CD43^{+} monocytes	0.0895 (ns)	0.0547 (ns)	0.0457 (*)
	Neutrophils	0.1021 (ns)	0.8274 (ns)	0.8849 (ns)
7 days post irradiation (dpi)	CD4 T cells	0.0105 (*)	0.0029 (**)	0.272 (ns)
	CD8 T cells	0.3928 (ns)	0.9542 (ns)	0.4929 (ns)
	NK cells	0.8264 (ns)	0.2477 (ns)	0.876 (ns)
	B cells	0.1277 (ns)	0.1154 (ns)	0.6913 (ns)
	His48^{+} monocytes	0.0061 (**)	0.0003 (***)	0.0363 (*)
	CD43^{+} monocytes	0.1523 (ns)	0.0475 (*)	0.244 (ns)
	Neutrophils	0.0573 (ns)	0.019 (*)	0.897 (ns)

Supplementary Figure 15. Impact of oxygen levels and irradiation modes on the circulating immune cell of glioma bearing rats, receiving 25 Gy. (a) CD4 T cells; (b) CD8 T cells; (c) NK cells; (d) B cells; (e) CD43^{high} His48^{low} monocytes; (f) CD43^{low} His48^{high} monocytes; (g) Neutrophils . hpi = hours post-irradiation, dpi = days post-irradiation. (h) Summary of two-way ANOVA statistical analysis of only irradiated rat blood samples separated by timepoint. The data are presented as the mean ± standard deviation (SD).
