# Supplementary material for: Oxygen supplementation in anesthesia can block FLASH effect and anti-tumor immunity in conventional proton therapy
Source: Commun Med (Lond). 2023 Dec 15;3:183. doi: 10.1038/s43856-023-00411-9 (PMC10724215; doi:10.1038/s43856-023-00411-9)
Supplement: Supplementary file 2 — Description of Additional Supplementary Files [file 43856_2023_411_MOESM2_ESM.pdf]

Description of Additional Supplementary Files

File Name: Supplementary Data 1
Description: Source data
