# Supplementary material for: Oxygen supplementation in anesthesia can block FLASH effect and anti-tumor immunity in conventional proton therapy
Source: Commun Med (Lond). 2023 Dec 15;3:183. doi: 10.1038/s43856-023-00411-9 (PMC10724215; doi:10.1038/s43856-023-00411-9)
Supplement: Supplementary file 4 — Reporting Summary [file 43856_2023_411_MOESM4_ESM.pdf]

nature portfolio

Corresponding author(s): Yolanda Prezado

Last updated by author(s): Oct 10, 2023

Reporting Summary

Nature Portfolio wishes to improve the reproducibility of the work that we publish. This form provides structure for consistency and transparency in reporting. For further information on Nature Portfolio policies, see our Editorial Policies and the Editorial Policy Checklist.

Statistics

For all statistical analyses, confirm that the following items are present in the figure legend, table legend, main text, or Methods section.

n/a	Confirmed	
☐	☒	The exact sample size (*n*) for each experimental group/condition, given as a discrete number and unit of measurement
☐	☒	A statement on whether measurements were taken from distinct samples or whether the same sample was measured repeatedly
☐	☒	The statistical test(s) used AND whether they are one- or two-sided<br>*Only common tests should be described solely by name; describe more complex techniques in the Methods section.*
☒	☐	A description of all covariates tested
☐	☒	A description of any assumptions or corrections, such as tests of normality and adjustment for multiple comparisons
☐	☒	A full description of the statistical parameters including central tendency (e.g. means) or other basic estimates (e.g. regression coefficient) AND variation (e.g. standard deviation) or associated estimates of uncertainty (e.g. confidence intervals)
☐	☒	For null hypothesis testing, the test statistic (e.g. *F*, *t*, *r*) with confidence intervals, effect sizes, degrees of freedom and *P* value noted<br>*Give P values as exact values whenever suitable.*
☐	☒	For Bayesian analysis, information on the choice of priors and Markov chain Monte Carlo settings
☒	☐	For hierarchical and complex designs, identification of the appropriate level for tests and full reporting of outcomes
☒	☐	Estimates of effect sizes (e.g. Cohen's *d*, Pearson's *r*), indicating how they were calculated

Our web collection on statistics for biologists contains articles on many of the points above.

Software and code

Policy information about availability of computer code

Data collection	N/A
Data analysis	N/A

For manuscripts utilizing custom algorithms or software that are central to the research but not yet described in published literature, software must be made available to editors and reviewers. We strongly encourage code deposition in a community repository (e.g. GitHub). See the Nature Portfolio guidelines for submitting code & software for further information.

Data

Policy information about availability of data

All manuscripts must include a data availability statement. This statement should provide the following information, where applicable:

- Accession codes, unique identifiers, or web links for publicly available datasets
- A description of any restrictions on data availability
- For clinical datasets or third party data, please ensure that the statement adheres to our policy

The raw data of flow cytometry and behavioral studies that support the findings of this study are available from the corresponding author, YP, upon reasonable request.

Human research participants

Policy information about studies involving human research participants and Sex and Gender in Research.

Reporting on sex and gender	N/A
Population characteristics	N/A
Recruitment	N/A
Ethics oversight	N/A

Note that full information on the approval of the study protocol must also be provided in the manuscript.

Field-specific reporting

Please select the one below that is the best fit for your research. If you are not sure, read the appropriate sections before making your selection.

☒ Life sciences ☐ Behavioural & social sciences ☐ Ecological, evolutionary & environmental sciences

For a reference copy of the document with all sections, see nature.com/documents/nr-reporting-summary-flat.pdf

Life sciences study design

All studies must disclose on these points even when the disclosure is negative.

Sample size	We determined the number of animals per group using G*power software.
Data exclusions	Behavioral tests data at 3 months post-irradiation for the 15 Gy series (in Supplementary Information) were excluded from the study. There were works done in the animal facility, so the controls didn't performed as expected and so we decide to eliminate the series.
Replication	The behavior studies presented in this article have not not been replicated, due to the limited access to the proton beam line for research. Concerning the studies on tumor-bearing rats, they were carried out in 3 independent experiments, which deemed to be reproducible.
Randomization	Bioluminescence imaging (BLI) using an IVIS spectrum (PerkerElmer) was used to verify the presence of the tumor before irradiation, and to allocate the animals to the different experimental groups. Animals were assigned to groups so that each experimental group had the same average bioluminescence signal. In cohort of healthy rats, rats were divided evenly between the experimental conditions.
Blinding	Behavioral analysis was performed blinded and condition revealed after analysis was completed. All histopathological assessments were performed blinded by a board-certified veterinary pathologist.

Reporting for specific materials, systems and methods

We require information from authors about some types of materials, experimental systems and methods used in many studies. Here, indicate whether each material, system or method listed is relevant to your study. If you are not sure if a list item applies to your research, read the appropriate section before selecting a response.

Materials & experimental systems

n/a	Involved in the study	
☐	☒	Antibodies
☐	☒	Eukaryotic cell lines
☒	☐	Palaeontology and archaeology
☐	☒	Animals and other organisms
☒	☐	Clinical data
☒	☐	Dual use research of concern

Methods

n/a	Involved in the study	
☒	☐	ChIP-seq
☐	☒	Flow cytometry
☐	☒	MRI-based neuroimaging

Antibodies

Antibodies used	Rat BD FcBlock (Purified Mouse Anti-Rat CD32, D34-485, 550273, BD Biosciences, RRID: AB_393570);<br>Mouse Anti-Rat CD4 (clone OX-35, 740256, BD Biosciences, RRID: AB_2740000);<br>Mouse Anti-Rat CD25 (clone OX-39, 748725, BD Biosciences, RRID: AB_2873129);

	Mouse Anti-Rat CD8a (clone OX-8, 740041, BD Biosciences, RRID:AB_2739811);<br>Mouse Anti-Rat CD3 (clone 1F4, 563949, BD Biosciences, RRID: AB_2738504);<br>Mouse Anti-Rat CD45 (clone OX-1, 740140, BD Biosciences, RRID: AB_2739896);<br>Mouse Anti-Rat CD161a (clone 10/78, 744053, BD Biosciences, RRID: AB_2741956);<br>Mouse Anti-Rat Itgrn AlpE2 (CD103) clone OX-62, 744679, BD Biosciences, RRID: AB_2742415);<br>Granulocyte Marker Monoclonal Antibody (His48) (clone HIS48, 11-0570-82, eBioscience, RRID: AB_465100);<br>Mouse Anti-Rat RT1B (clone OX-6, 745824, BD Biosciences, RRID: AB_2743273);<br>Rat Anti-Rat CD49d (clone MRα4-1, 130-111-297, Miltenyi Biotec, RRID: AB_2658497);<br>Human anti-Rat CD43 (clone REA503, 130-107-684, Miltenyi Biotec, RRID: AB_2658083);<br>Rat Anti-Rat CD45R (B220) (clone HIS24, 25-0460-82, eBioscience, RRID: AB_2573351);<br>anti-rat CD43 (clone W3/13, 1614070, SONY Biotechnology; RRID: Not available);<br>Mouse Anti-Rat CD11b/c (clone OX-42, 752230, BD Biosciences, RRID: Not available).
Validation	Antibodies were only used if validated by the manufacturer via their website.<br>Use of Rat BD FcBlock (Purified Mouse Anti-Rat CD32, D34-485, 550273, BD Biosciences, RRID: AB_393570) is routinely tested for flow cytometry by BD Biosciences.<br>Mouse Anti-Rat CD4 (clone OX-35, 740256, BD Biosciences, RRID: AB_2740000) is routinely tested for flow cytometry by BD Biosciences.<br>Mouse Anti-Rat CD25 (clone OX-39, 748725, BD Biosciences, RRID: AB_2873129) is qualified for flow cytometry by BD Biosciences.<br>Mouse Anti-Rat CD8a (clone OX-8, 740041, BD Biosciences, RRID:AB_2739811) is qualified for flow cytometry by BD Biosciences.<br>Mouse Anti-Rat CD3 (clone 1F4, 563949, BD Biosciences, RRID: AB_2738504) is routinely tested for flow cytometry by BD Biosciences.<br>Mouse Anti-Rat CD45 (clone OX-1, 740140, BD Biosciences, RRID: AB_2739896) is qualified for flow cytometry by BD Biosciences.<br>Mouse Anti-Rat CD161a (clone 10/78, 744053, BD Biosciences, RRID: AB_2741956) is qualified for flow cytometry by BD Biosciences.<br>Mouse Anti-Rat Itgrn AlpE2 (CD103) clone OX-62, 744679, BD Biosciences, RRID: AB_2742415) is routinely tested for flow cytometry by BD Biosciences.<br>Granulocyte Marker Monoclonal Antibody (His48) (clone HIS48, 11-0570-82, eBioscience, RRID: AB_465100) is routinely tested for flow cytometry by eBiosciences (thermofisher) at a dilution of 0.25 µg/test.<br>Mouse Anti-Rat RT1B (clone OX-6, 745824, BD Biosciences, RRID: AB_2743273) is qualified for flow cytometry by BD Biosciences.<br>Rat Anti-Rat CD49d (clone MRα4-1, 130-111-297, Miltenyi Biotec, RRID: AB_2658497) is tested by Miltenyi Biotec for flow cytometry at a dilution of 1/50 (QC tested).<br>Human anti-Rat CD43 (clone REA503, 130-107-684, Miltenyi Biotec, RRID: AB_2658083) is tested by Miltenyi Biotec for flow cytometry at a dilution of 1/10 (QC tested).<br>Rat Anti-Rat CD45R (B220) (clone HIS24, 25-0460-82, eBioscience, RRID: AB_2573351) is tested for flow ctometry by eBiosciences (Thermofischer) at 0,5 µg/test.<br>anti-rat CD43 (clone W3/13, 1614070, SONY Biotechnology; RRID: Not available): Each lot of this antibody is quality control tested by immunofluorescent staining with flow cytometric analysis. For flow cytometric staining, the suggested use of this reagent is ≤0.06 µg per million cells in 100 µl volume.<br>Mouse Anti-Rat CD11b/c (clone OX-42, 752230, BD Biosciences, RRID: Not available) is qualified for flow cytometry by BD Biosciences.

Eukaryotic cell lines

Policy information about cell lines and Sex and Gender in Research

Cell line source(s)	RG2-luc, a rat glioma cell line RG2 [D74] (ATCC® CRL-2433™) transfected with the GFP and luciferase genes
Authentication	Procedure of authentification is not possible for murine cell lines at our institute
Mycoplasma contamination	Cells were tested negative for mycoplasma by pPCR
Commonly misidentified lines<br>(See ICLAC register)	No commonly misidentified cell lines were used

Animals and other research organisms

Policy information about studies involving animals; ARRIVE guidelines recommended for reporting animal research, and Sex and Gender in Research

Laboratory animals	Fischer rats 344 males
Wild animals	The present study did not use wild animals.
Reporting on sex	Males
Field-collected samples	The present study did not use field-collected samples.
Ethics oversight	All experiments were approved and authorized by the French Ministry of Research under the reference APAFIS no. 2021033117587802 and 2022040609163783.

Note that full information on the approval of the study protocol must also be provided in the manuscript.

Flow Cytometry

Plots

Confirm that:

☒ The axis labels state the marker and fluorochrome used (e.g. CD4-FITC).

☒ The axis scales are clearly visible. Include numbers along axes only for bottom left plot of group (a 'group' is an analysis of identical markers).

☒ All plots are contour plots with outliers or pseudocolor plots.

☒ A numerical value for number of cells or percentage (with statistics) is provided.

Methodology

Sample preparation

Blood was collected on tumor-bearing rats receiving 25 Gy in tubes with ethylene diamine tetra-acetic acid (EDTA) (OZYME), 24 hours and 7 days post-irradiation. Red blood cells were lysed using a Red Blood Cell Lysis Solution (Miltenyi Biotec). Cells were then stained.

At 8 days post-irradiation, tumors and the contralateral hemisphere of the rat brain were dissected and incubated in digestion solution containing Dulbecco's phosphate-buffered saline (DPBS), 1 mg/mL collagenase D (Roche), 0.1 mg/mL DNase I (Sigma), and 3% fetal calf serum (FCS). Tissues were then mechanically disrupted to obtain single cell suspensions in DPBS with 0.5% bovine serum albumin (BSA) and 2 mM EDTA. Cells were mixed with 30% isotonic Percoll Solution, centrifuged, and blocked with anti-CD32 (FcγRII) blocking agent. Cells were then stained.

Cells staining: cells were incubated in a viability stain at a 1:1000 dilution (FVS780, BD Biosciences, RRID: AB_2869673) and immunolabeled in buffer containing PBS and 3% of fetal bovine serum (Table S2). Counting beads were added to each sample before flow cytometry (CountBright™ Plus Absolute Counting Beads, Thermo Fisher).

Instrument

BD LSRFortessa™ X-20 (BD Life Sciences)

Software

Software for data collection was BD FACSDiva™ (BD Life Sciences), the software for data analysis were FlowJo™ v10.6 (BD Life Sciences) and GraphPad Prism 9 (GraphPad Software, CA, United States)

Cell population abundance

The samples were not FACS-sorted.

Gating strategy

Gating strategy employed in the flow cytometry analysis of the tumor: cells of interest were selected on SSC-A vs FSC-A, doublets were excluded on FCS-W vs FCS-A. Dead cells were excluded with FVS-e780. Among live cells, cells positive for the YFP and negative of CD11b/c were gated as tumor cells (RG2) and were excluded from the analysis. Among RG2neg cells, CD45+ cells were gated. Among CD45+ cells, CD3+ and CD3neg cells were gated on CD11b/c-AF700 vs CD3-BV605. Among CD3+ cells, CD8 T cells and CD4 T cells were gated on CD8-BV421 vs CD4-BUV395. Among CD8 T cells, TRM were gated as positive to CD103-BV785. Among CD4 T Cells, Treg were gated as positive to CD25-BUV737.
Among CD3neg cells, CD161high cells and NK excluded cells were gated on His48-FITC vs CD161-BV711. Among CD161high cells, NK cells were gated on CD8-BV421 vs Rt1b-BB700 as CD8+ and Rt1bneg.
Among NK excluded, B excluded and B220high cells were gated on His48-FITC vs B220-PECy7. Among B220high cells, B cells were gated on CD8-BV421 vs Rt1b-BB700 as CD8neg and Rt1b+.
Among B excluded cells, CD103neg cells and cDC1 cells were gated on RT1b-BB700 vs CD103-BV785. Among CD103neg cells, CD11b/c+ cells were gated on CD11b/c-AF700vsCD43-APC. Among these cells, CD8+ macrophages cand CD8neg cells were gated on CD8-BV421 vs CD49d-PE. Among CD8neg, CD49d+ cells and CD49neg myeloid cells were gated on His48-FITC vs CD49d-PE. Among CD49d+ cells, His48+CD43neg monocytes/macrophages cells, Neutrophils and CD43+ His48neg monocytes were gated on His48-FITC vs CD43-APC. (The gating strategy is represented in Figure S1)

Gating strategy employed in the flow cytometry analysis of the brain: Among all events, cells of interest were gated on SSC-A vs FSC-A. Among these cells, doublets were excluded on FCS-W vs FCS-A. Dead cells were excluded with FVS-e780. Among live cells, cells positive for the GFP and negative of CD11b/c were gated as tumor cells (RG2) and were excluded from the analysis (<1 %). Among RG2neg cells, CD45low and CD45+ cells were gated on CD11b/c-AF700 vs CD45-BV510. Among CD45 + cells, microglia (CD49dneg cells) was gated on His48-FITC vs CD49d-PE as negative for both markers. Among microglia, the expression of Rt1b+ was verified on SSC-A vs RT1b-BB700. (The gating strategy is represented in Figure S2)

Gating strategy employed in the flow cytometry analysis of the blood: Among all events, cells of interest were gated on SSC-A vs FSC-A. Dead cells were excluded with FVS-e780. Among these cells, doublets were excluded on FCS-H vs FCS-A. Among singlets, CD45+ cells were gated on CD45-BV510 vs FSC-A. Among CD45+ cells, CD3+ and CD3neg cells were gated on CD3-BV605 vs FSC-A. Among CD3+ cells, CD8 T cells and CD4 T cells were gated on CD8-BV421 vs CD4-BUV395. Among CD3neg cells, NK cells and CD161neg cells were gated on CD161-BV711 vs His48-FITC. Among CD161neg cells, B cells and B220neg cells were gated on B220-PECy7 vs CD161-BV711. Among B220neg cells, Neutrophils and SSC-Alow cells were gated on His48-FITC vs SSC-A. Among SSC-Alow cells, CD43low His48high monocytes and CD43high His48low monocytes were gated on His48-FITC vs CD43-APC. (The gating strategy is represented in Figure S3).

☒ Tick this box to confirm that a figure exemplifying the gating strategy is provided in the Supplementary Information.

Magnetic resonance imaging

Experimental design

Design type	N/A, we realized only anatomical MRI and Gd injected MRI
Design specifications	N/A
Behavioral performance measures	N/A

Acquisition

Imaging type(s)	Structural
Field strength	7
Sequence & imaging parameters	(i) Morphological T2-weighted (T2W) images with a repetition time (TR) of 2500 ms and 4 echo times (TE) of 11, 33, 55 and 77 ms. A signal averaging of 2 was employed. In all, 21 slides were acquired in a total time of 10 min 40 s.<br>(ii) T1-weighted (T1W) TurboRare sequences with a TR of 800 ms and a TE of 6.05 m. A signal averaging of 4 was employed. A total of 21 slides were acquired in a time of 5 min 7 s. Four acquisitions were performed, one before and three (5, 10 and 15 min) after the intravenous injection of a bolus of 100 µmol/kg Gd-DOTA (Guerbet SA, Villepinte, France).<br>(iii) T1 fast low-angle shot (FLASH) sequences with a TR and TE of 114.89 and 3.1 ms, respectively. A flip angle of 30° and a signal averaging of 4 were used. A total of 9 slides were acquired in a total time of 1 min 28 s. Acquisitions were made just before and 5, 10, and 15 min after the intravenous injection of a bolus of 100 µmol/kg Gd-DOTA (Guerbet SA, Villepinte, France).
Area of acquisition	Whole brain. The field of view was 35 mm × 35 mm, the in-plane resolution amounted to 0.14 mm × 0.14 mm, and the slice thickness and gap were 0.8 and 0.3 mm, respectively.

Diffusion MRI ☐ Used ☒ Not used

Preprocessing

Preprocessing software	Paravision 6.0.1, Bruker, Inc.
Normalization	Not normalized
Normalization template	Not normalized
Noise and artifact removal	Paravision 6.0.1 "motion averaging module" was used to minimize micro movements due to breathing. No respiratory trig. No cardiac trig.
Volume censoring	The measurement volume is defined to have the best signal to noise ratio. The fied of view is greater than the diameter of the measurement antenna. The measurement antenna is homogeneous over its length (Bird cage 3.2 cm in diameter and about 4 cm long). These conditions were tested on an agar gel phantom and verified on animals.

Statistical modeling & inference

Model type and settings	N/A, we realized only anatomical MRI and Gd injected MRI to vizualized brain tissue injuries
Effect(s) tested	N/A, we realized only anatomical MRI and Gd injected MRI to vizualized brain tissue injuries

Specify type of analysis: ☒ Whole brain ☐ ROI-based ☐ Both

Statistic type for inference (See Eklund et al. 2016)	N/A, we realized only anatomical MRI and Gd injected MRI to vizualized brain tissue injuries
Correction	N/A, we realized only anatomical MRI and Gd injected MRI to vizualized brain tissue injuries

Models & analysis

n/a	Involved in the study
☒	☐ Functional and/or effective connectivity
☒	☐ Graph analysis
☒	☐ Multivariate modeling or predictive analysis
